# Chiral Morphing of Liquid Crystal Polymer Networks

**DOI:** 10.1002/advs.77074

**Published:** 2026-08-13

**Authors:** Laurens T. de Haan

**Affiliations:** ^1^ Guangdong Provincial Key Laboratory of Optical Information Materials and Technology Institute of Electronic Paper Displays South China Academy of Advanced Optoelectronics South China Normal University Guangzhou China; ^2^ SCNU‐TUE Joint Lab of Device Integrated Responsive Materials (DIRM) National Center for International Research On Green Optoelectronics South China Normal University Guangzhou China

**Keywords:** chirality, liquid crystal polymers, shape morphing, soft actuators, stimuli‐responsiveness

## Abstract

Liquid crystal polymer networks represent a fascinating and promising class of soft materials due to their inherent stimuli‐responsive contraction and expansion, the direction of which is determined by the alignment director. This unique feature makes liquid crystal networks a prime candidate for the development of chiral actuators, and this topic has seen a marked increase in interest during recent years. This review brings together the various studies on macroscopic chiral shape morphing in liquid crystal polymer networks and actuators. First, it discusses the theoretical and fundamental studies that demonstrate how the shape selection arises from the anisotropy, geometric misorientation and aspect ratio of liquid crystal polymer ribbons. Then, it summarizes the different ways in which chiral shape morphing can be achieved, including bilayers, mechanical shaping, and 3D printing. Then it gives some examples of the exciting potential applications that have been developed for these systems, including function actuators inspired by plant tendrils and flowers and soft robotics applications.

## Graphical abstract

This paper reviews how macroscopic chirality spontaneously arises from liquid crystal polymer materials. It describes how alignment is utilized to create the mechanical anisotropy, geometric misorientation and asymmetry that leads to chiral shape morphing, how shape selection between helicoids and helices arises, the techniques used to prepare these devices, and finally shows several exciting applications.

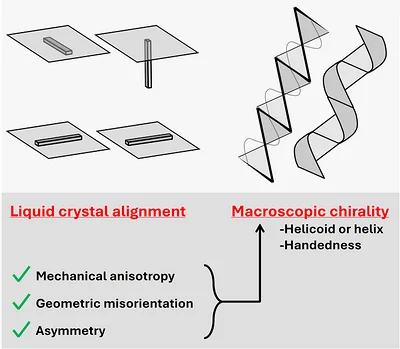

## 1 Introduction

**FIGURE 1:**
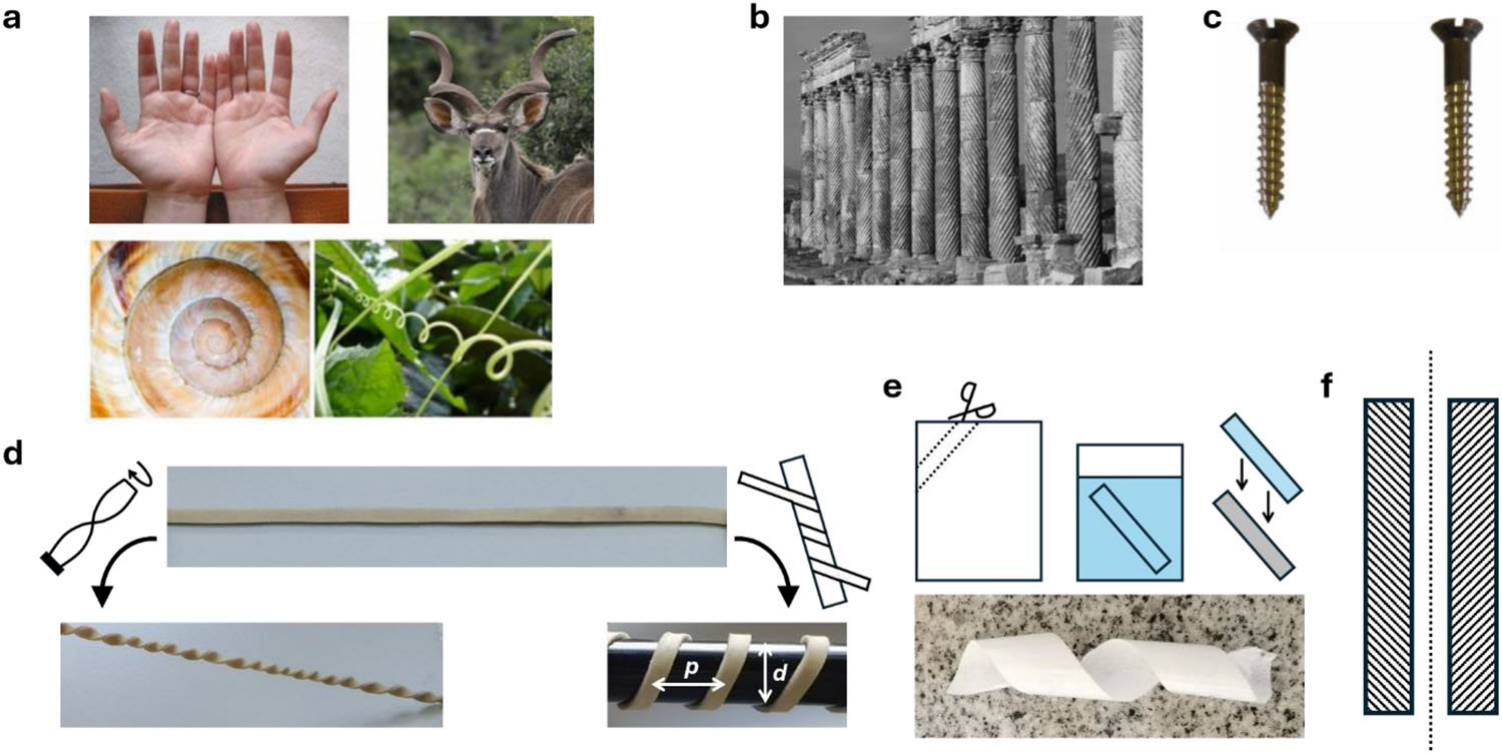
(a) Examples of chirality in nature. Top: human hands, helix‐shaped horns. Reproduced with permission [6]. Copyright 2013, Springer Nature. Bottom: Helix shell and plant tendril. Adapted under the terms of the CC‐BY Creative Commons Attribution 4.0 International license [2]. Copyright 2022, Wiley‐VCH. (b) Helical fluted columns of the Cardo Maximus of Apamea. Reproduced with permission [5]. Copyright 2016, Taylor and Francis Group. (c) Screws with typical helical grooves. The right‐handed screw is the standard variant, the left‐handed screw is almost never produced. Reproduced with permission [6]. Copyright 2013, Springer Nature. (d) Deformation of a rubber band into chiral shapes. Left: the rubber band is held on one side and the other side is twisted, which results in a helicoid ribbon. Right: The rubber band is wound around the shaft of a pen, which results in a helical ribbon. Photos were taken by the author. (e) Experiment illustrating the spontaneous formation of a helical ribbon from a flat bilayer consisting of wet paper and plastic tape. Because the paper consists of aligned fibers and is cut at an acute angle, it swells anisotropically. After it is combined with the plastic tape and dried, the anisotropic deswelling leads to the formation of a helical ribbon. Photo taken by the author. (f) Illustration showing how flat ribbons cut at either a positive angle or a negative angle are enantiomorphs without possessing a 3D chiral shape. The diagonal lines visualize the alignment of the paper fibers.

Though we may not always realize it, chiral shapes are ubiquitous in the world around us. Helices and spirals are commonly found in nature, for example in our hands, animal horns, shells, and plant tendrils(Figure 1a) [1, 2, 3, 4]. Humans have been inspired by these shapes and have been incorporating them in design and art for centuries (Figure 1b) [5]. However, these shapes can also serve practical purposes, such as in helix staircases, springs, screws (Figure 1c) [6], the rifling of gun barrels, and propellers. The functions of these shapes are varied: in some cases they are simply aesthetically pleasing, but they can also break symmetry (such as in pairs of appendages and horns), provide improved mechanical and structural properties (such as in springs and tendrils), or turn rotational motion into forward motion (such as in screws, gun barrel rifling and propellers). While in scientific writing the concept of chirality usually applies to molecular structures, it also applies to geometric figures of macroscopic size that cannot be superimposed onto their own mirror image, as originally defined by Lord Kelvin in 1894 [7]. In fact, the word chirality is derived from the Greek word for hand, which is itself a macroscopic chiral object. In this review, the geometric definition of chirality is used unless otherwise specified.

3D chiral shapes can be produced from nonchiral flat ribbons by mechanically deforming them in two basic ways [8] (Figure 1d). The first method is to hold the ribbon on one end and rotate the other end to produce a twisted helicoid ribbon, which requires stretching of the material. The second method is to wind the ribbon around a rod, either real or imaginary, to produce a helical ribbon, which requires bending of the material. The main difference between a helicoid and a helical ribbon is the center line: in the former the center line is straight and the sides of the ribbon twist around it, while in the latter both the center line and the sides follow a helical path around the central axis. Both shapes can be defined by three parameters: handedness, pitch and diameter (with the handedness often being expressed as the sign of the pitch value). For helicoids, the handedness is determined by whether the direction of rotation is clockwise or counterclockwise, the pitch is determined by the length of the ribbon divided by the number of rotations, and the diameter is determined by the width of the ribbon. For helical ribbons, the handedness is determined by the sign of the bending direction, the pitch is determined by the angle of the bending, and the diameter by the magnitude of the bending, with the latter being equivalent to the diameter of the (real or imaginary) cylinder. A ribbon can also have an in‐between shape with characteristics of both a helicoid and a helix, and a spectrum of shapes exists from a pure helicoid to a pure spiral [9].

An intriguing possibility is to make a 3D chiral shape arise spontaneously from a flat, seemingly nonchiral ribbon, a process that is driven by a combination of mechanical anisotropy and geometric misorientation [10]. A simple but illustrative experiment can easily be performed at home (Figure 1e). A ribbon is diagonally cut from a sheet of paper and soaked in water, causing it to swell. This swelling is anisotropic due to the alignment of the fibers of the paper. A piece of plastic tape is then stuck to the back of the wet ribbon and cut to match its size. When the paper ribbon dries, it undergoes shrinking in the same direction as the initial swelling, which provides mechanical anisotropy, while the diagonal cutting provides geometric misorientation. Morphing into a 3D chiral shape requires asymmetry between the two faces of the ribbon, which in this bilayer system is provided by the difference in deswelling capability between the paper layer and the plastic tape. The handedness of the final ribbon is determined by the sign of the cutting angle, while the pitch and diameter are determined by the value of the cutting angle and the magnitude of the swelling.

While at first glance it seems as if in the previously mentioned bilayer the chirality of the helix arises spontaneously, this is not the case! The ribbons cut at a positive and negative acute angle of the same value are mirror images of one another due to the anisotropy of the fibers and can therefore be considered chiral objects even before deformation has occurred (Figure 1f). These two enantiomorphs produce helical ribbons of opposite handedness upon drying. The transformation from a flat ribbon to a helical ribbon is driven by the mechanical mismatch between the paper layer and plastic layer, which induces bending. This simple experiment shows that deformation into a chiral shape can be programmed into a flat object by making use of anisotropy. This can also be accomplished using different materials systems, as long as they are capable of undergoing a spontaneous, directed mechanical deformation [11, 12].

**FIGURE 2:**
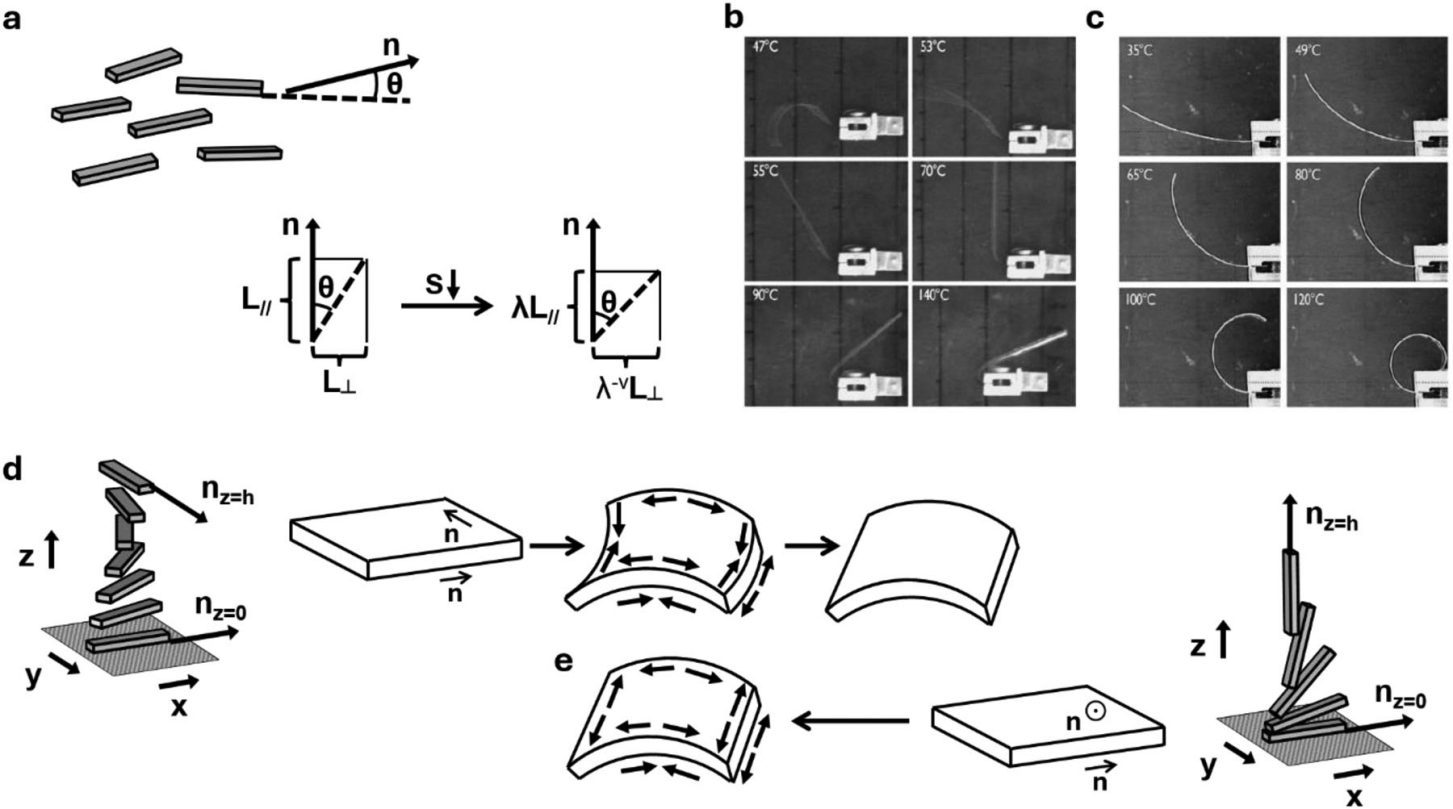
(a) Mechanism of the anisotropic deformation of a liquid crystal polymer network upon reduction of the order parameter. Here n is the alignment director, θ is the average deviation from the alignment director, S is the order parameter, and **L_//_** and **L_┴_** are the lengths parallel and perpendicular to the alignment director, respectively. Reproduced with permission. Copyright 2014, Elsevier [28]. (b) A liquid crystal polymer ribbon with twisted nematic alignment bends upon heating. Reproduced with permission [35]. Copyright 2005, Wiley‐VCH. (c) A similar ribbon with splayed nematic alignment bends upon heating. Reproduced with permission [35]. Copyright 2005, Wiley‐VCH. (d) Mechanism of deformation of a film with twisted nematic alignment. Upon heating the material contracts parallel the alignment director and expands perpendicular to it. This causes anticlastic bending, until the increasing stretches cause suppression of curvature in one direction. (e) Mechanism of deformation of a film with splayed nematic alignment. In this case, bending only takes place in the direction of the alignment director on the planar face of the film.

An interesting class of anisotropic materials is based on crosslinked liquid crystal (LC) polymers [13]. Such polymers are prepared from liquid crystal monomers, also known as reactive mesogens. Reactive mesogens are fluids consisting of rod‐shaped molecules that are aligned along a common director, which is called the alignment director. This alignment can be fixed in a solid material when the monomers are both polymerized and crosslinked, which can be done either simultaneously or sequentially. When the nematic order of a liquid crystal polymer material is disrupted by heating, it will anisotropically deform by contracting along the alignment director and expanding perpendicular to it (Figure 2a) [14, 15, 16, 17]. These spontaneously deforming materials are often called liquid crystal actuators due to their potential to do useful work. The effect also takes place in the opposite direction, where increasing the order parameter of the material leads to contraction perpendicular to the alignment director and expansion parallel to it. The spontaneous deformation can also be achieved in other ways, for example by UV‐irradiation when photo‐responsive molecules such as azobenzenes are incorporated in the network [18, 19, 20, 21, 22, 23] or by swelling a hygroscopic material with water [24, 25, 26].

Using alignment techniques such as alignment layers, a great deal of control can be exerted over the alignment geometry of the solid material, which can be monodomain, multidomain, and even vary in the plane and/or through the thickness of a polymer film. This combination of alignment‐dependent deformation and alignment control allows researchers to program the deformation of these materials in a predictable manner [27, 28, 29, 30]. Compared to in‐plane deformations, bending out of plane is even more interesting. Bending of flat sheets takes place when the deformation on one side of the sheet is different compared to the other side, and this symmetry breaking can be achieved in two ways. The first method is by applying the stimulus to only one side of the flat sheet, causing only that side to change its shape while the other side is unaffected [31, 32]. A more exciting possibility is to program the out‐of‐plane deformation into the material itself, so it arises even if the stimulus is applied evenly. To do so, usually one side of the material needs to respond to the stimulus in a different way compared to the other side. As demonstrated by the example in Figure 1e, an effective way to accomplish this is by preparing bilayers, and this method is also applicable to liquid crystal actuators [33, 34].

A more elegant way to achieve bending deformation in liquid crystal actuators is to make sure the alignment director of the mesogens is different between the two faces of the film. This allows bending deformations to be truly programmed into the supramolecular structure of a liquid crystal polymer film by utilizing twisted alignment (Figure 2b,d) or splayed alignment Figure 2c,e) [35, 36, 37, 38]. In the case of twisted alignment, the alignment director rotates smoothly in plane between the two faces of the film. In most examples the alignments at the top and bottom faces are at a 90° angle, which causes the alignment directors at both faces of the film to be orthogonal to each other. When a liquid crystal polymer ribbon with 90° twisted alignment is heated, one surface contracts along the long axis of the ribbon and expands along the short axis, while the opposite surface expands along the long axis and contracts along the short axis. These opposing deformations lead to bending in two directions which is are orthogonal and of opposite sign, also called anticlastic deformation, which leads to a saddle‐like shape and a change from zero Gaussian curvature to negative Gaussian curvature. However, a change in Gaussian curvature leads to large stretches in the material as the bending in one direction causes parts of the film to be far removed from the neutral plane associated with bending in the other direction [39]. As such, at larger curvatures the bending in one direction is suppressed in favor of bending exclusively in the other direction, and the deformation becomes monoclastic. In a ribbon it tends to be more favorable to suppress the bending in the width direction, causing it to bend only along its length axis.

In ribbons with splayed alignment the director rotates smoothly from planar at one face of the film to homeotropic at the other face. When such a ribbon is heated, the face with planar alignment contracts in one direction in the plane and expands perpendicular to that direction, while the homeotropic face expands in all directions in the plane. The expansions do not cause bending, so the film undergoes monoclastic bending regardless of the magnitude of the deformation [35, 36]. The bending direction is always along the alignment director on the planar side, and the planar side always ends up on the inside of the bend. The Gaussian curvature stays zero throughout the bending process, so this deformation is essentially stretch free, and the response to temperature is greater compared to ribbons of the same dimensions and material with twisted alignment.

As may be expected based on the title of this review, stimuli‐responsive deformation of liquid crystal polymer films into chiral shapes has garnered extensive interest, as chiral shape deformations provide a relatively straightforward but nonetheless interesting and valuable way to achieve stimuli‐responsive morphing of flat ribbons into 3D shapes. Analogous to the bilayer ribbon shown in Figure 1e, the alignment of the mesogens and the corresponding directed shape deformation provide mechanical anisotropy, while the geometric misorientation can be realized by aligning the mesogens at an acute angle with the long axis of the ribbon. Finally, it is required to break the mechanical symmetry between the two faces of the ribbon to achieve out‐of‐plane deformation.

The alignment‐dependent existence of two distinct enantiomorphs of one polymer film adds an additional dimension to these systems, and the chiral shapes have additional potential beyond the more traditional stretching and bending materials. Deformation of flat ribbons into helicoids, or from one helicoid shape into a different one, produces rotational motion that could be useful in various devices. Helix formation of liquid crystal ribbons can be used as a grasping or rolling motion in soft robotics. This review aims to demonstrate the ways in which chiral deformations can be programmed into liquid crystal polymer materials and the mechanisms through which they deform, followed by a summary of demonstrated practical examples and applications. Section 2 discusses the mechanisms of chiral shape morphing in monolithic liquid crystal polymer ribbons. Section 3 shows the various ways in which chiral actuators can be designed and prepared, and finally section 4 gives some examples of how these actuators can be developed towards practical applications.

To properly discuss the topic of chiral shape morphing in liquid crystal polymer materials, it is necessary to properly define some terminology. In both practical experiments and theoretical simulations, the liquid crystal polymer film often exists in the form of a ribbon, which is a shape that is very flat and has a distinct long and short axis in the plane. These ribbons can be deformed into chiral shapes by either twisting or bending, and the resulting shapes are both often called a helix, with the latter often being referred to as a coil or spiral. To semantically distinguish between these two forms, from this point onward the twisted shape will be referred to as a “helicoid”, and the bent shape will be called a “helical ribbon”.

Many of the materials discussed have twisted nematic alignment, and it is important to distinguish between the handedness of nematic twist on a molecular level and the handedness of the macroscopic shape that arises due to this alignment. When discussing twisted nematic alignment geometry, it is often necessary to mention the angle between the long axis of the ribbon and the alignment director at the surface of the film, defined here as the alignment offset angle θ. The sign of this angle is particularly important, and the sign depends on the direction from which the surface is viewed. Therefore, θ will be defined as the angle between the long axis of the ribbon and the surface alignment director when looking towards that surface. This means that the alignment geometry of a ribbon with 90° twisted nematic alignment and θ = 45° or θ = ‐45° can be described with a single value of θ due to the rotational symmetry. When θ is rotated clockwise from the long axis of the ribbon it has a negative value, and when it is rotated counterclockwise it has a positive value.

## 2 Chiral Shape Transitions of Monolithic Liquid Crystal Polymer Ribbons

### 2.1 Deformation of Flat Ribbons Into Chiral Shapes

As established in the introduction, the formation of chiral shapes from flat ribbons is driven by mechanical anisotropy and geometric misorientation. In the case of liquid crystal polymer ribbons, the mechanical anisotropy arises from the alignment of the mesogens, and the geometric misorientation is between the alignment director and the long axis of the ribbon. As such, to obtain a flat liquid crystal polymer ribbon which changes into a chiral shape the alignment director of the mesogens needs to be in a direction that is neither parallel nor perpendicular to the long axis of the ribbon, while there should also be some sort of asymmetry between the two faces of the ribbon to facilitate bending. Achieving asymmetry in the stimulus is difficult in this case as the chiral deformation tends to expose the other side of the ribbon to the stimulus.

Compared to applying an asymmetric stimulus, programming the shape change into the material is a better option, and bilayers, twisted nematic alignment, and splayed nematic alignment can all be utilized for this purpose. As mentioned in the introduction, flat ribbons can be deformed into chiral shapes by either twisting into a helicoid or bending into a helical ribbon. When a ribbon is twisted, the Gaussian curvature changes from zero to negative, while bending does not change the Gaussian curvature [39]. Besides the distinction between helicoids and helical ribbons, there is the distinction between left‐handed chirality and right‐handed chirality to consider. This means there are four basic chiral shapes a liquid crystal polymer ribbon can deform into: a left‐handed helicoid, a right‐handed helicoid, a left‐handed helix, and a right‐handed helix. As discussed in the introduction, the handedness of any chiral shape morphing is determined by the angle between the anisotropic deformation and the long axis of the ribbon. Therefore, in liquid crystal polymer ribbons the handedness is determined by the sign of the alignment offset angle θ.

**FIGURE 3:**
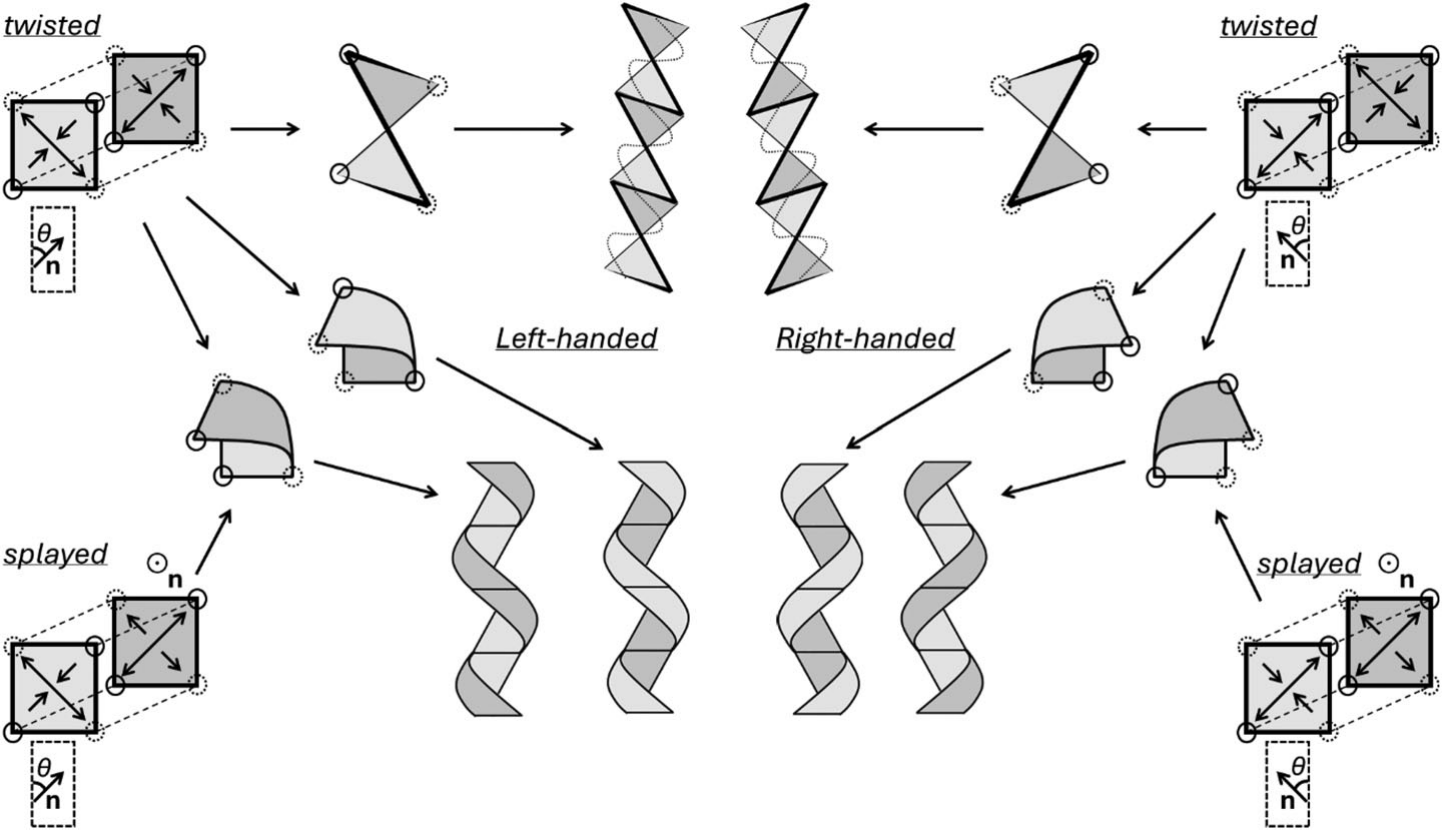
Schematic overview showing the chiral deformation of LC polymer ribbons with twisted and splayed alignment. The two faces of the film and the contraction and expansion direction in each face are shown. In films with splayed alignment one face has planar alignment and the other face has homeotropic alignment (indicated as ⨀_n_). Ribbons with this alignment can only form helical ribbons, the homeotropic side always ends up on the outside of the helix and the planar side on the inside, and the handedness is determined by the sign of θ. In films with twisted alignment the director rotates 90° from one side to the other side. These films can form helicoids or helical ribbons, the selection of which is determined by whether the curvature is suppressed, which is in turn mostly determined by the aspect ratio of the ribbon. Due to the rotational symmetry, when a helical ribbon is formed either face can end up on the inside/outside. The handedness of either shape is determined by the sign of θ.

In monolithic liquid crystal polymer ribbons, twisted and splayed alignment can both be utilized to create the asymmetry between the faces needed for chiral shape deformation. As liquid crystal polymer ribbons with splayed alignment only undergo monoclastic bending upon heating, their Gaussian curvature does not change, and they can only adopt helical ribbon shapes. The direction of bending is easy to determine, as it is determined by the angle θ between the long axis of the ribbon and the alignment director on the surface with planar alignment. If θ is negative the result is a left‐handed helix, and if it is positive the result is a right‐handed helix. The planar side of such ribbons always ends up on the inside of the helix, as it is the only side with in‐plane contraction, and the homeotropic side is always on the outside (Figure 3). On the other hand, in ribbons with twisted nematic alignment the deformation is anticlastic. These ribbons can form either helicoids or helices, depending on whether the curvature along one axis is suppressed or not.

The shape selection of liquid crystal polymer ribbons with twisted nematic alignment has been carefully studied [40, 41, 42, 43, 44]. The main parameter determining the shape selection between a helicoid or helix is the width‐to‐thickness ratio of the ribbon, with narrow ribbons forming helicoids and broader ribbons forming helices. Ribbons with a small width‐to‐thickness ratio are easier to be stretched than to be bended and form helicoids, while ribbons with a large width‐to‐thickness ratio are easier to be bended than to be stretched, which causes suppression of curvature along one axis which results in helix ribbons. Both computer simulations and laboratory experiments have confirmed this behavior. Unlike in ribbons with splayed alignment, in ribbons with 90° twisted alignment the two faces of the film have rotational symmetry if θ at the surface is 45° or ‐45°, which means theoretically either of them can end up on the inside of a helix ribbon. Like in ribbons with splayed alignment, the handedness of the helicoid or helix is solely determined by the sign of θ: If this angle is negative, the shape will be left‐handed, and if it is positive, the shape will be right‐handed (Figure 3).

A temperature change is the most straightforward stimulus to lower the order parameter and drive the deformation of chiral liquid crystal actuators [45, 46, 47, 48]. However, irradiation with UV light has become a particularly popular stimulus due to the ease of application and removal. Azobenzenes are the logical choice to serve as the photo‐responsive moiety [49], but UV‐responsive molecular motors have also been utilized [50, 51]. Chiral actuators containing hygroscopic moieties can undergo shape deformations depending on humidity [52, 53], and other moieties allow chiral deformation when different chemical stimuli are applied [54, 55]. Achieving water‐responsiveness usually requires treating the material with a base or an acid to generate a hygroscopic polymer salt. Performing this treatment on only one side of the ribbon can induce asymmetry without requiring twisted or splayed alignment, allowing chiral bending deformations from materials with uniaxial alignment geometries.

### 2.2 Winding, Unwinding and Handedness Change

**FIGURE 4:**
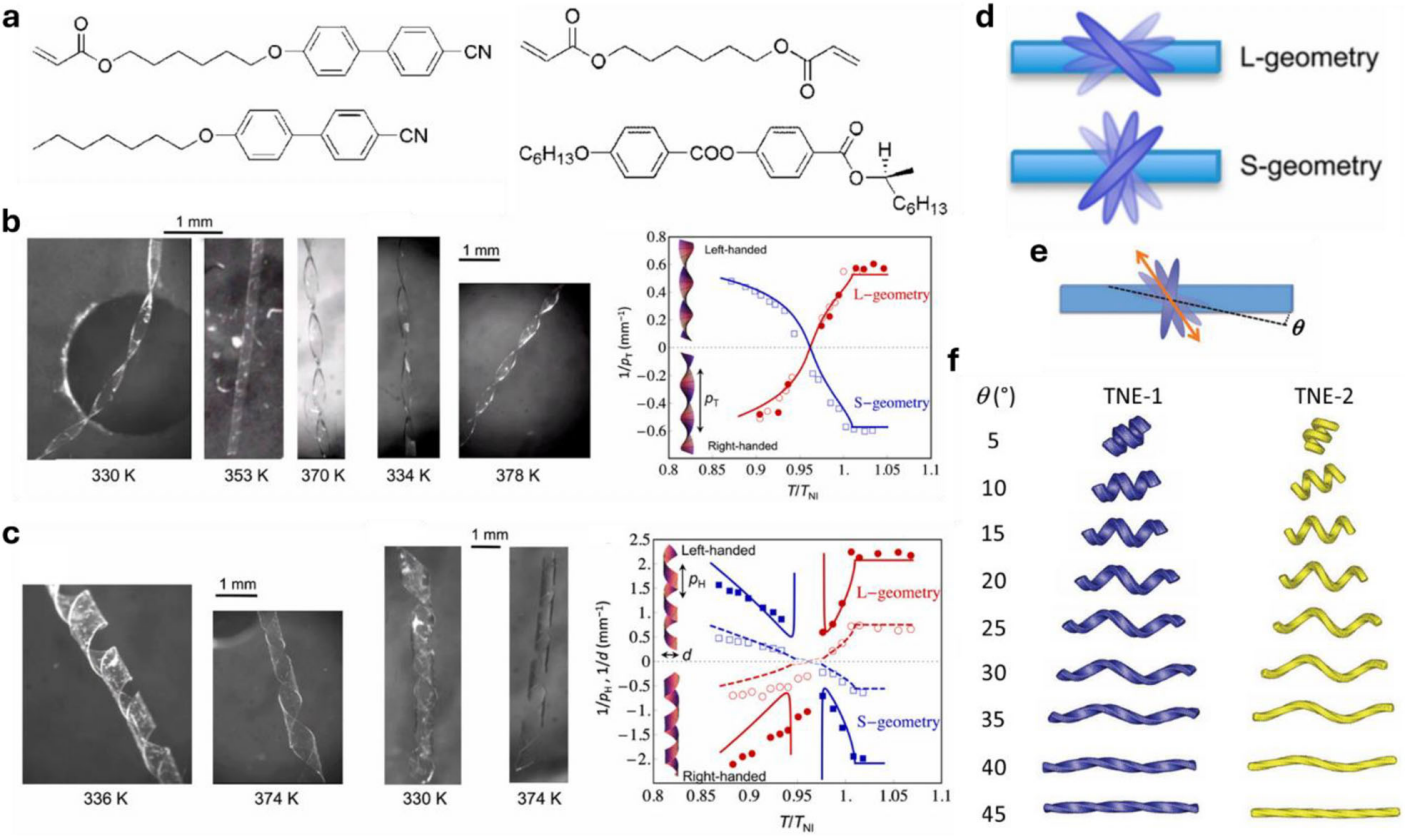
(a) Liquid crystal mixture used to prepare chiral ribbons with twisted nematic alignment. The mixture consists of a reactive mesogen, a crosslinker, a nonreactive mesogen, and a chiral dopant. (b) Narrow ribbons have helicoid shapes at room temperature. Upon heating, the helicoids unwind until they are flat, then wind up into helicoids of opposite handedness. (c) Broader ribbons are shaped like a helical ribbon at room temperature. These also unwind and rewind upon heating. (d) Schematic depiction of the L‐ and S‐geometry of ribbons with twisted nematic alignment. (e) Schematic depiction of X‐geometry in ribbons with twisted nematic alignment. (f) Computer simulations showing the relationship between ribbon shape and θ in ribbons with X‐geometry. At small angles the shape is a helical ribbon, and as the angle is increased the shape gradually changes towards a helicoid shape. Figures reproduced from Sawa et al.  [40]. Copyright 2011, National Academy of Sciences.

The previous section introduced the deformation of flat liquid crystal polymer ribbons into helicoids and helical ribbons. Besides these shape transformations, there is a strong scientific interest in ribbons that have a chiral initial shape and then change into a different shape upon application of a stimulus. An early set of well known experiments were performed using liquid crystal polymer films with twisted nematic alignment in which the used liquid crystal monomer mixture contains unreactive liquid crystals, which can later be removed from the crosslinked material (Figure 4a) [40]. Removal of the unreactive liquid crystals results in anisotropic shrinkage in the direction perpendicular to the alignment director, so ribbons cut from these films have helicoid or helix shapes at room temperature. For example, a ribbon with a negative angle θ has a right‐handed helicoid or helix shape at room temperature, while a positive value of θ leads to left‐handed shape. When heated, the expansion also takes place in the direction perpendicular to the alignment director, causing these films to first unwind to become flat and then form the helicoid or helical ribbon shape of the opposite handedness.

The previously mentioned experiments clearly show the effect of aspect ratio of the ribbons on the shape selection, with narrower ribbons forming helicoids (Figure 4b) and broader ribbons forming helices (Figure 4c) [40]. In these experiments, the alignment geometry is defined by the angle between the long axis of the film and the so‐called midplane director, which is the alignment director halfway between the two surfaces of the film. When the midplane director is parallel to the long axis of the ribbon it is described as L‐geometry, and when it is perpendicular it is described as S‐geometry (Figure 4d). As the chiral dopant used in these experiments induces a left‐handed twist in the molecular orientation, ribbons with L‐geometry have a positive angle θ, and in ribbons with S‐geometry this angle is negative. It is important to keep in mind that if the nematic twist would be right‐handed this relation would be reversed, with L‐geometry ribbons having a negative θ and S‐geometry a positive θ. Using this information, the handedness of the chiral shapes after shrinkage and after heating can be predicted using the schematic shown in Figure 3. Interestingly, whether the ribbons had L‐ or S‐geometry was found to have some effect on the shape selection, with the critical width for shape selection being smaller for ribbons with L‐geometry compared to those with S‐geometry, and helices with L‐geometry being slightly tighter wound.

Besides L‐ and S‐geometry, off‐axis geometries of networks with twisted nematic alignment were investigated both in practical experiments and theoretical simulations [56, 57]. These differ from the previously mentioned geometries in that the angle θ at both faces of the ribbon is not 45° or ‐45°, or in other words, the midplane director is neither parallel nor perpendicular to the long axis of the ribbon. This is referred to as off‐axis geometry or X‐geometry (Figure 4e). A consequence of this is that the two faces of the film no longer have rotational symmetry, so the offset angle θ is different for each face, though it is of the same sign. The geometry of ribbons with X‐geometry can be described in terms of the angle θ which is most closely aligned with the long axis of the ribbon, or in other words, the angle with the smallest absolute value. Like in the work described in the previous paragraph, the networks contained a nonreactive component that was washed out after polymerization, so the resulting ribbons were strongly deformed at room temperature.

It was found that ribbons with X‐geometry do not have the ability to form true helicoids even at aspect ratios that would normally result in such a shape [56, 57]. The explanation for this is that the central line in a helicoid is straight, which requires the midplane director to be symmetric, as is the case only for the L‐ and S‐geometries. Computer simulations were performed to show the effect of θ on the shape selection of ribbons with a narrow width (Figure 4f). At values of θ close to 0°, the angle was found to strongly affect the pitch of the helices but to have no effect on the helix diameter. When the angle was increased towards 45° the shape started to more and more resemble a helicoid, though as mentioned a true helicoid could only be observed at 45°. Interestingly, these simulations also showed that when the angle is moved from positive to negative, the angle at which the shape transitions from right‐handed to left‐handed, and was nonchiral, was not 0° but ‐2.5°. This is due to a small contribution of the twist handedness of the twisted nematic alignment. Experiments confirmed these results.

**FIGURE 5:**
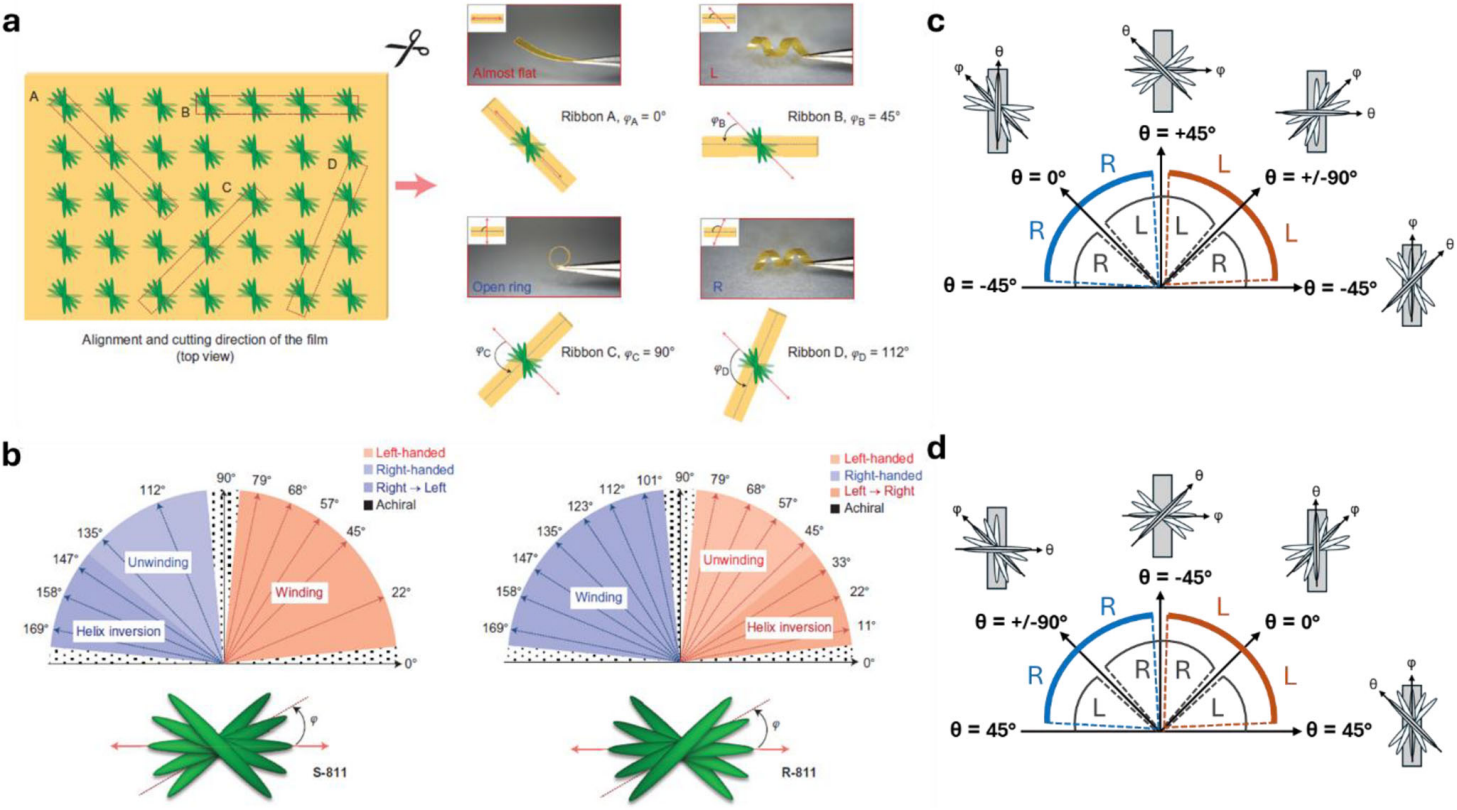
Cutting angles of light‐responsive ribbons from a liquid crystal polymer film containing azobenzene groups and unreactive mesogens. These ribbons immediately deform after being removed from the film without any stimulus being applied. Reproduced with permission [58]. Copyright 2014, Springer Nature. (b) Initial shape and shape deformation upon UV irradiation of the ribbons. The figure on the left is for films with a left‐handed nematic twist, and the figure on the right for films with a right handed nematic twist, as is determined by the choice of chiral dopant. The blue and red fans indicate the chirality of the initial shape, and the blue and red text indicates the effect of UV irradiation on the shape. Reproduced with permission [58]. Copyright 2014, Springer Nature. (c) Comparison of the handedness of the initial shape of the ribbons compared to the expected chirality based the alignment director at both faces of the ribbons, assuming anisotropic shrinkage. The red and blue wedges indicate the actual chirality, while the gray wedges indicate the expected chirality. Both ϕ and θ, as well as their directions relative to each other, are indicated for four different ribbons. These ribbons have a left‐handed nematic twist. (d) Same as figure c, but for ribbons with a right‐handed nematic twist.

Light‐responsive winding, unwinding and helix inversion was also reported [58, 59]. This was achieved by incorporating an azobenzene reactive mesogen into a network with twisted nematic alignment. Like in the previous examples, unreactive liquid crystals were added to the reactive mesogen mixture, but in this case they were not removed after UV polymerization. The experiments were performed using two different chiral dopants, S‐811 and R‐811, that resulted in a left‐handed twist and a right‐handed twist of the nematic director through the thickness, respectively. Ribbons were cut from the films at various angles, and they immediately deformed into helical ribbons (Figure 5a). In this work, the alignment geometry of the ribbons was defined using the angle ϕ between the midplane director and the long axis of the ribbon (Figure 5b). This causes the value of the offset angle θ to be determined by the combination of ϕ and the handedness of the nematic twist. For example, when ϕ = 0° and the nematic twist is left‐handed, θ = ‐45°, but when ϕ = 0° and the nematic twist is right‐handed, θ = +45°.

However, when comparing the values of ϕ and θ to the expected shapes as shown in Figure 3, it becomes clear that the shape selection for the ribbons after deswelling is different from what is expected: ribbons with θ = +45° and θ = ‐45°deform into nonchiral shapes, while ribbons with an angle θ of 0° deform into helices [58, 59]. This behavior was caused by the formation of a polymerization gradient across the thickness of the ribbon: during photopolymerization the UV light is stronger on the irradiated side, which causes the polymerizable molecules to diffuse towards that side and away from the shaded side. This was proven by the fact that the irradiated side always ended up on the outside of the bend, which indicates a stronger contraction on the nonirradiated side due to the presence of a larger amount of unreactive mesogen. It seems that the presence of the gradient causes the contraction direction to be offset by 45° when the nematic twist is left‐handed and ‐45° when it is right‐handed, or in other words, the contraction is perpendicular to the midplane director (Figure 5c,d). This indicates that the material on the shaded side contains too little polymer to participate in the deformation. Upon UV‐irradiation, the ribbons would either show winding, unwinding, or helix inversion (Figure 5b). This seems to be determined by the alignment director on the irradiated side: if it is aligned closer to the long axis of the ribbon, unwinding/inversion is observed, and if it is aligned closer to the short axis winding is observed.

Besides twisted nematic alignment, light‐responsive helical ribbons based on different alignment geometries were reported [60]. By applying electric fields to a nematic liquid crystal mixture containing a chiral dopant followed by UV‐polymerization, films with surface frustrated lying helix and Helfrich‐Hurault patterns were prepared. The resulting films had line patterns that were visible with both bright field and polarized optical microscopy. Like in the previous example, the reactive mesogen mixture contained unreactive mesogens that caused a crosslink gradient through the film thickness and azobenzene mesogens to enable a UV response. The relationship between the ribbon cutting angle with respect to the rubbing direction of the alignment layers and the shape selection was investigated, and was found to be different for films with surface frustrated lying helix alignment compared to those with a Helfrich‐Hurault alignment.

**FIGURE 6:**
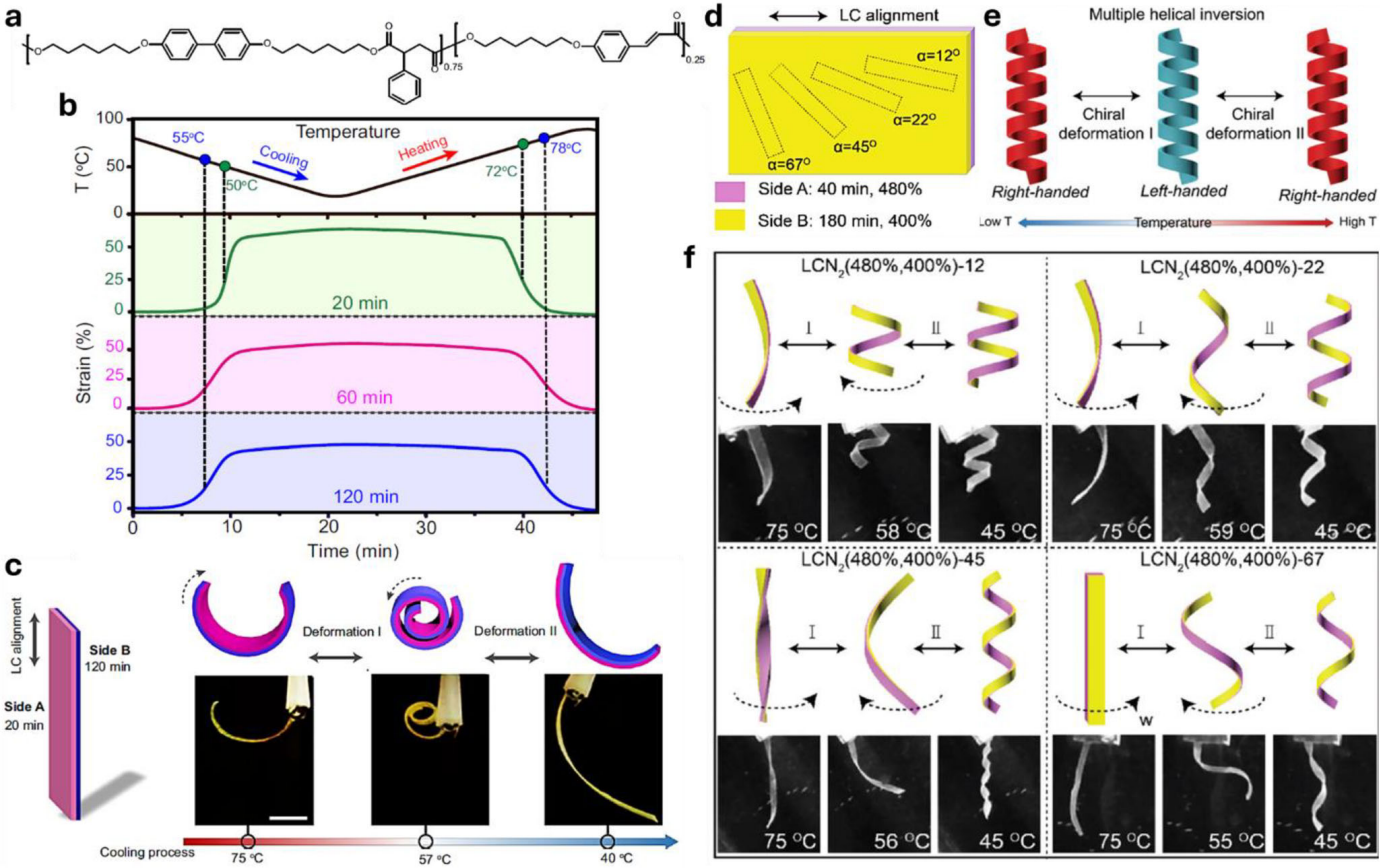
Molecular structure of the crosslinkable liquid crystal polyester with hexyl spacers and cinnamate groups. Adapted under the terms of the CC‐BY Creative Commons Attribution 4.0 International license [61]. Copyright 2021, Springer Nature. (b) Thermal deformation behavior of films with various UV‐crosslinking times. Adapted under the terms of the CC‐BY Creative Commons Attribution 4.0 International license [61]. Copyright 2021, Springer Nature. (c) Thermal deformation of a ribbon with a less crosslinked face A and a more crosslinked face B. At high temperature the film bends towards face A, and at low temperatures it bends towards face B. Adapted under the terms of the CC‐BY Creative Commons Attribution 4.0 International license [61]. Copyright 2021, Springer Nature. (d) Ribbons cut from the asymmetric film at various angles. Adapted with permission [62]. Copyright 2021, The Royal Society of Chemistry. (e) Schematic representation helix inversion of chiral ribbons from the asymmetric film at various temperatures. Copyright 2021, The Royal Society of Chemistry. (f) Shapes of the chiral ribbons with several different cutting angles at low, medium and high temperatures. Copyright 2021, The Royal Society of Chemistry.

A different way to achieve thermal helix inversion was reported as well [61, 62]. In this case, a biphenyl polyester with hexyl spacers, which was polymerized using phenylsuccinic acid as the chain extender, was used to prepare the liquid crystal networks (Figure 6a). The polymer had incorporated cinnamate moieties that could be used to crosslink it under UV irradiation to fix the alignment, which was performed after the liquid crystal molecules had been uniaxially aligned by stretching. It was found that the deformation behavior upon temperature change was different for different crosslinking times. Upon cooling, the films with longer crosslinking times started to deform at a higher temperature and they deformed more gradually as the temperature was lowered, while the films with shorter crosslink times started deforming at a lower temperature and deformed over a shorter temperature range and finally to a larger extent (Figure 6b).

The previously mentioned effect was used to introduce asymmetry into the films by irradiating one side for a short time and the other side for a longer time [61, 62]. This led to films that initially bent strongly towards the side with a short crosslinking time as the temperature was lowered, then bent towards the side with a long crosslinking time at lower temperatures (Figure 6c). The asymmetry could be further amplified by additionally stretching the film after the first crosslinking step. By cutting ribbons at various angles with respect to the alignment director (Figure 6d), films could be made that deformed into helices. Due to the asymmetry between the two sides, which caused one face or the other to be the outside face of the helix at different temperatures, helix inversion could be achieved (Figure 6e,f). It is interesting to observe that in this work the asymmetry between the faces is not created by using the alignment geometry, and is instead induced using different UV light doses for each side during UV curing.

An interesting system has been reported in which a multidomain liquid crystal polymer ribbon showed deformation into a helix shape upon heating [63]. The polymers were synthesized using functionalization of the polybutadiene (PB) block of a thermoplastic styrene−butadiene−styrene (SBS) triblock copolymer with mesogenic groups through click and esterification reactions. Some of the mesogenic groups that were added to the polymer backbone were chiral to induce a cholesteric phase. The material was cast from solution to obtain the ribbons without any alignment of the mesogens. Surprisingly, when heated to isotropic these ribbons would undergo a deformation into helix shapes, the handedness of which was linked to the handedness of the cholesteric helix of the polymer. In fact, the handedness of the helices appeared to be of opposite handedness compared to the handedness of the cholesteric helix. Achiral and racemic polymers produced no helix shapes upon heating, and stretching the ribbons also led to the loss of the helix formation. It seems that in this system the molecular chirality and macroscopic chiral deformation are linked, though it remains unclear how the forces that cause the formation of the helices arise.

## 3 Design and Preparation of Chiral Liquid Crystal Actuators

### 3.1 Chiral Actuators Based on Bilayers

While the liquid crystal polymer ribbons with twisted and splayed alignment reported in the previous section provide an elegant way to achieve chiral deformations in monolithic ribbons, some progress has been reported using bilayer systems that is very much worth mentioning. Some works report chiral shape transformations by combining a liquid crystal polymer film with a second polymer film of a different material. For example, an LC film was combined with a polystyrene layer [64]. An aligned liquid crystal elastomer film was prepared using a two‐step crosslinking method, and the polystyrene layer was attached using a film‐transfer technique. By depositing the polystyrene film onto a ribbon of the liquid crystal elastomer by film‐transfer, a bilayer was created that deformed into a helicoid upon heating. In a different study, an azobenzene‐containing reactive mesogen mixture was spray coated onto a thermoplastic PET film and polymerized [65]. The resulting bilayer could then be thermally shaped into helical ribbons due to the thermoplastic properties of the PET layer, which were UV‐responsive thanks to the azobenzene groups. Two different kinds of helical ribbon were prepared: those with the LC layer on the inside would wind tighter upon UV exposure, while those with the LC layer on the outside would unwind.

**FIGURE 7:**
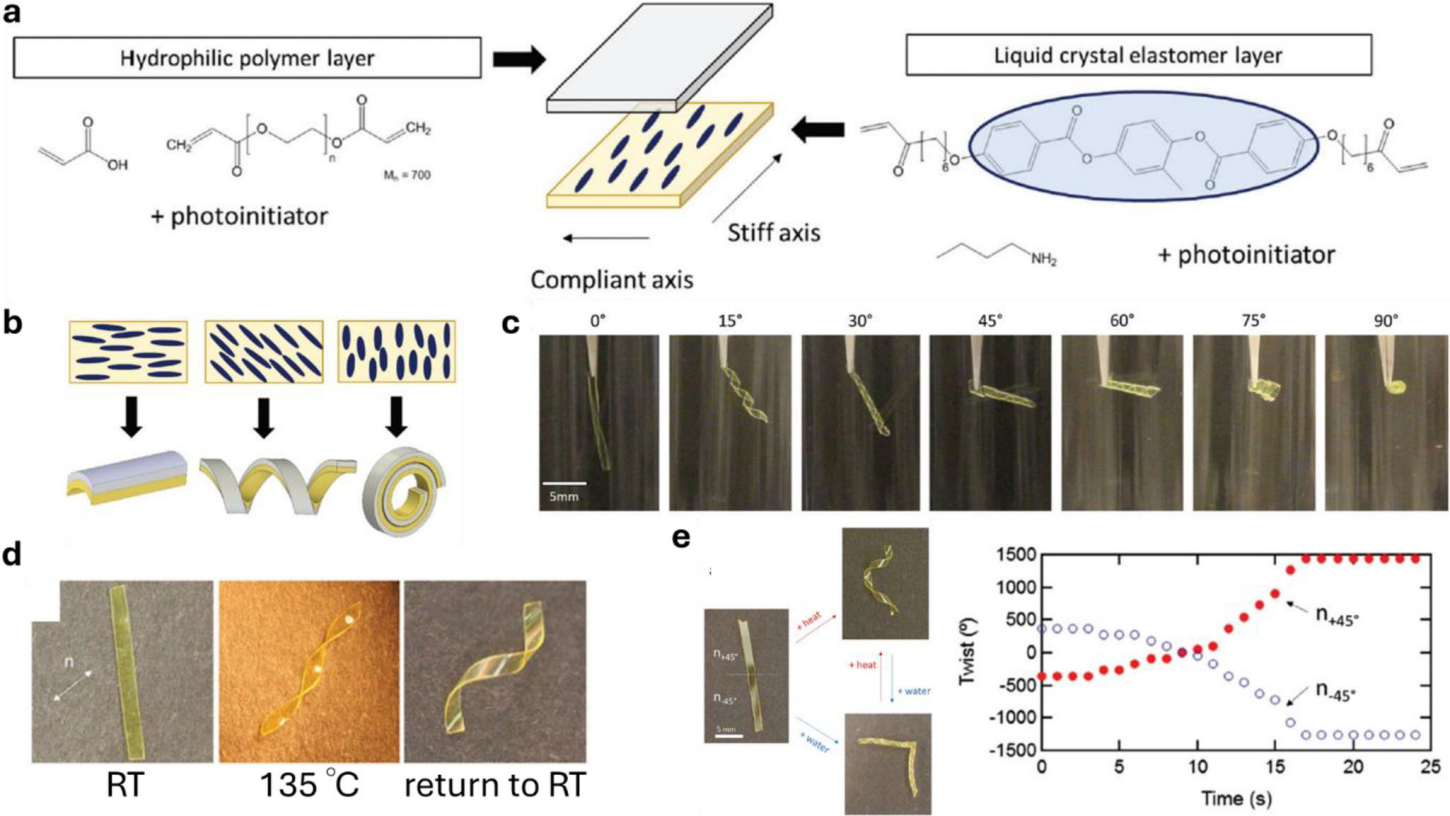
(a) Materials and preparation methods for dual‐responsive bilayers of a hydrophilic polymer film and a liquid crystal elastomer film. The two films are prepared separately and combined using a photocurable adhesive. (b) Schematic depiction of the effect of the alignment director of the liquid crystal elastomer film on the shape selection of the bilayer upon immersion in water. The liquid crystal layer is yellow and always ends up on the inside of the resulting 3D shape. (c) Bilayers with various angles between the alignment director and the long axis of the ribbon upon deformation by immersion in water. (d) Temperature response of the bilayer. The initially flat ribbon changes to a helicoid upon heating to 135°C. Upon cooling back to room temperature, the ribbon changes from a helicoid to a helical ribbon of the same handedness and stays in that shape. (e) A photopatterned ribbon with one half having the angle between the alignment director and the long axis of the film at +45° and the other half at ‐45°. When the ribbon is exposed to either heat or water, both sides change into helices of opposite handedness, and the handedness of these helices is reversed upon switching the stimulus from one to the other. Adapted with permission [66]. Copyright 2017, The Royal Society of Chemistry.

In a different example, a liquid crystal polymer film was combined with a water‐responsive hydrophilic poly(acrylic acid) film using a photocurable adhesive (Figure 7a) [66]. When the hydrophilic layer was swollen with water, its expansion drove the bending of the bilayer towards the liquid crystal polymer side. The bending direction was guided by the alignment director of the liquid crystal polymer layer (Figure 7b,c), as the elastic modulus of this layer was approximately five times greater along the alignment director compared to perpendicular to the alignment director, causing bending takes place perpendicular to the alignment director. Photopatterning of the alignment director was used to create various complex chiral shapes. Because the liquid crystal film is temperature‐responsive, the bilayers show dual‐responsiveness to water and heating (Figure 7d). When heated to 135°C, which is above the T_g_ of the poly(acrylic acid), the bilayer ribbon deformed into a helicoid due to the deformation of the liquid crystal film, but now with the opposite handedness of the helix it formed when exposed to water. Further cooling caused the shape to change from a helicoid to a helix ribbon of the same handedness. It stayed in that shape at room temperature because as the film was cooled past the T_g_ of the hydrophilic layer the modulus of this layer increased, locking the chiral shape. The handedness inversion could be further demonstrated by switching from heating to water immersion (Figure 7e).

**FIGURE 8:**
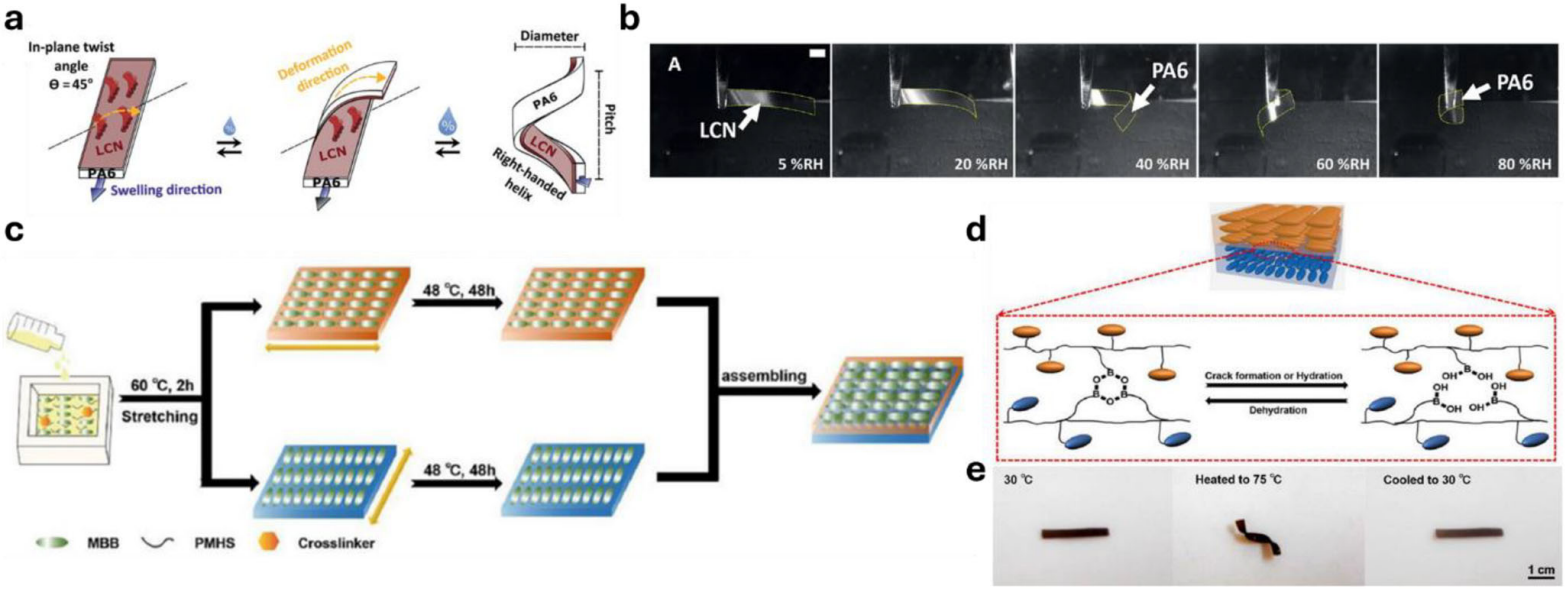
(a) Bilayer actuator consisting of a polyamide film and a liquid crystal polymer film. Upon exposure to water the polyamide film swells, causing a bending deformation with the liquid crystal film on the inside and the polyamide film on the outside. The alignment director at the surface of the LC film directs the bending towards a helical shape. Reproduced with permission [67]. Copyright 2018, Royal Society of Chemistry. (b) Shape change of a polyamide/liquid crystal network bilayer ribbon upon increasing the relative humidity. Reproduced with permission [67]. Copyright 2018, Royal Society of Chemistry. (c) Preparation procedure of a bilayer consisting of two liquid crystal polymer films with orthogonal alignment directors, which are attached to each other using exchangeable boroxine groups. Reproduced with permission [69]. Copyright 2020, Elsevier. (d) Schematic showing the reversible boroxine groups that keep the bilayer together. Reproduced with permission [69]. Copyright 2020, Elsevier. (e) Deformation of the bilayer into a helix upon heating. Reproduced with permission [69]. Copyright 2020, Elsevier.

Another way to achieve a water‐responsive bilayer system of a non‐liquid crystalline polymer film and a liquid crystal polymer network is through spray coating the liquid crystal monomer mixture onto a polyamide film (Figure 8a) [67]. A mixture of liquid crystal monomers was dissolved in xylene and sprayed on stretched polyamide using a spray gun and cured through UV polymerization. The stretched polyamide acted as an alignment layer for the liquid crystals. By adding a small, well‐controlled amount of chiral dopant to the liquid crystals a twisted alignment could be achieved without using alignment cells. When the bilayer was exposed to humidity, the polyamide side swelled, predominantly along the long axis of the ribbon. The liquid crystal alignment at the surface directed the deformation into a helix shape with the polyamide on the outside and the liquid crystal network on the inside (Figure 8b). Varying the amount of chiral dopant allowed some control over the shape selection of the helices by directing the alignment at the surface of the liquid crystal layer.

Besides combining a liquid crystal polymer film with a different polymer to make a bilayer, it is also possible to combine two liquid crystal polymer films to achieve bilayers with chiral shape deformations. In this case, orthogonal alignment directors of the layers cause a bending deformation in a way similar to monolithic films with twisted nematic alignment without requiring chirality on the molecular and/or supramolecular level. An inventive way to combine two liquid crystal films into a bilayer system makes use of exchangeable bonds. In one example of this technique, disulfide bonds were used for this purpose [68]. Monoacrylate liquid crystal monomers were uniaxially aligned and polymerized in the presence of a diacrylate crosslinker with an internal disulfide bond. Two of these films could then be combined orthogonally into a bilayer using a phosphine‐based catalyst solution which activated the disulfide exchange. Ribbons cut from these bilayers at an angle of ±45° deformed into helices upon heating. Similarly, two polysiloxane liquid crystal elastomers with boroxine groups could be combined into a bilayer actuator (Figure 8c and d) [69]. These films can be welded together at moderate temperatures due to an exchange reaction between the boroxine groups. By orthogonally welding two uniaxially aligned films a temperature‐responsive bending actuator could be realized, and by cutting this actuator at an angle of +45° a ribbon was created that deformed into a right‐handed helix when heated to the isotropic state (Figure 8e).

### 3.2 Chiral Actuators Made by Mechanical Shaping

Traditionally, liquid crystal polymer networks have been prepared as thin films, utilizing alignment layers to induce the monodomain alignment of the mesogens. However, a marked downside of these systems is that they are difficult to upscale. A potentially more widely applicable technique to create liquid crystal polymer materials with reversible chiral shapes is through mechanical shaping [70, 71, 72, 73, 74]. This technique requires a transition from a pliable form that can easily be deformed into a solid form that fixes the new shape, for example through covalent or noncovalent crosslinking or exchangeable bonds. To achieve liquid crystal polymer networks with chiral shapes the material is molded into that shape, either by rolling it around a cylinder to obtain a helix or by twisting it along the central axis to obtain a helicoid (analogous to the example in Figure 1d). The shear forces that arise during the molding process align the mesogenic molecules in the direction of the shear. In the next step the alignment needs to be fixed, for example by chemical or physical crosslinking or by crystallization. When the freestanding chiral object is heated or the order parameter is decreased in a different way, the contraction along the alignment director and expansion perpendicular to it work in the reverse direction compared to the initial shaping process, and the chiral shape unwinds and returns to the flat state. When actuators are made using mechanical shaping, the exact alignment profile of the liquid crystal molecules is often not known.

**FIGURE 9:**
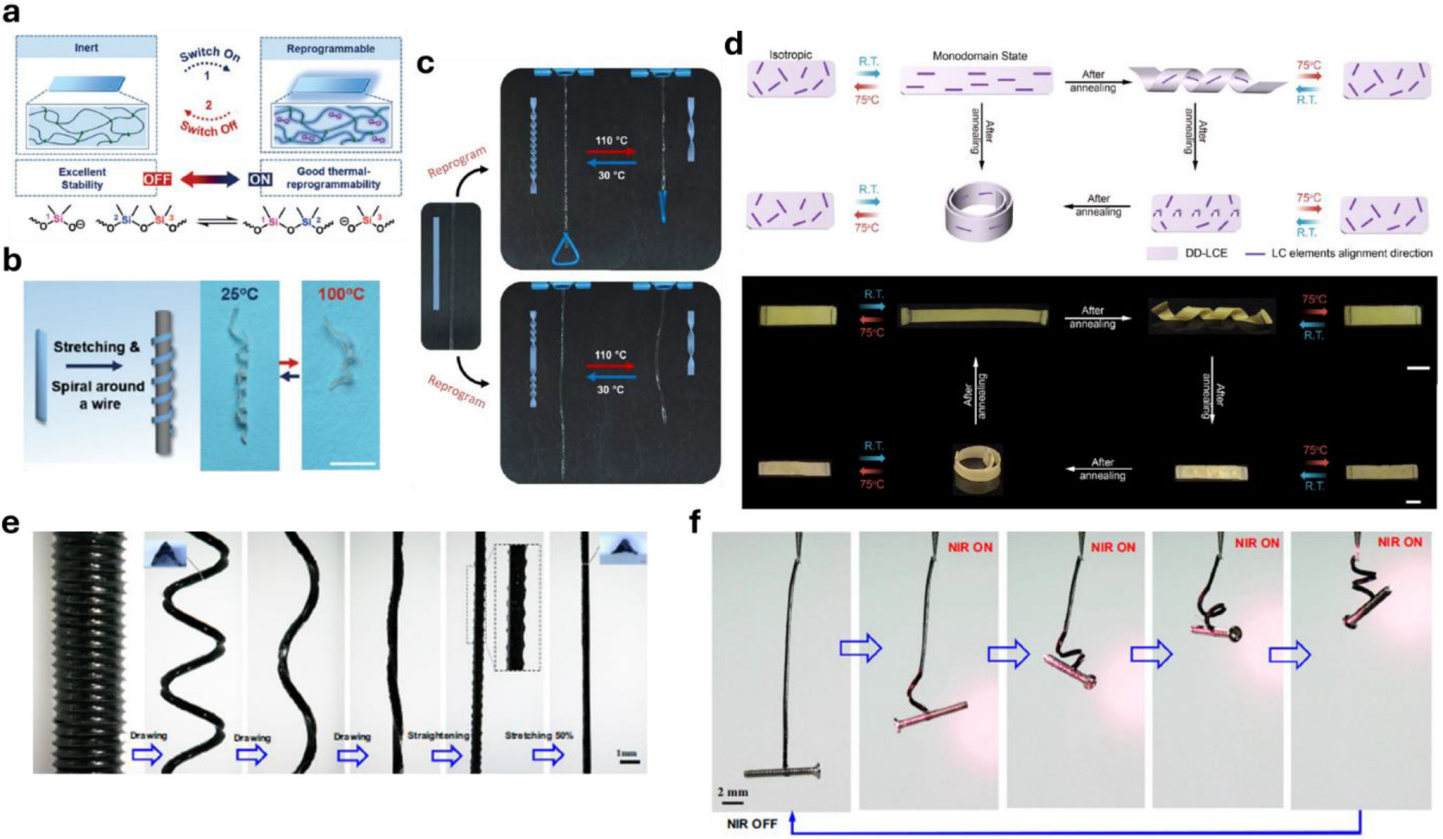
(a) Mechanism of the reshaping of a liquid crystal polysiloxane network. Reproduced with permission [71]. Copyright 2020, Wiley‐VCH. (b) Mechanical shaping of a liquid crystal polysiloxane network and the actuation behavior of the helix actuator by heating. Reproduced with permission [71]. Copyright 2020, Wiley‐VCH. (c) Reversible temperature response of helicoid ribbons prepared by the mechanical shaping of of polythiourethane‐liquid crystal polymer segmented copolymers. Reproduced under the terms of the CC‐BY Creative Commons Attribution 4.0 International license [72]. Copyright 2022, Wiley‐VCH. (d) Mechanical shaping and reversible deformation of liquid crystal polymer films with hydrogen‐bonding MAEUP groups and An‐A reversible covalent crosslinks. Reproduced with permission [73]. Copyright 2025, Wiley‐VCH. (e) Chiral shaping method where a helical fiber was prepared using a screw mold and then stretched into a straight fiber and crosslinked. Reproduced under the terms of the CC‐BY Creative Commons Attribution 4.0 International license [78]. Copyright 2021, Springer Nature. (f) Deformation of the straight fiber into a chiral shape upon IR heating. Reproduced under the terms of the CC‐BY Creative Commons Attribution 4.0 International license [78]. Copyright 2021, Springer Nature.

In one example of mechanical shaping to prepare chiral shapes, films and fibers of a liquid crystal elastomer were prepared using a diacrylate reactive mesogen and a tetrathiol chain extender through a base‐catalyzed Michael reaction [70]. The films and fibers were wound around a glass rod to deform them into a helix. Using an excess of the acrylate caused some acrylate groups to remain present after the reaction, which could be used to further crosslink the material through UV‐initiated free‐radical polymerization to permanently fix the shape. These helix shapes reversibly unwound upon heating them to the isotropic state. In a different example, a liquid crystal polysiloxane network was used for the shaping process [71]. In a post‐synthesis approach the siloxane network was swollen with a solution of the catalyst TMA‐DMSiO in chloroform to activate a dynamic siloxane exchange reaction that allowed reshaping of the material (Figure 9a). The material was then simultaneously stretched and wound around an iron wire and immobilized, and the network reconfiguration was thermally induced (Figure 9b). The reaction was stopped by heating to 150°C to decompose the catalyst and obtain the stable liquid crystal polymer helix. This helix would reversibly contract upon heating and cooling.

Besides covalent bonds, hydrogen bonding is a useful noncovalent interaction that can be used for chiral shaping. For example, segmented copolymers with hydrogen bonded polythiourethane segments and liquid crystal soft segments to obtain a thermoplastic material that could be reshaped at high temperatures [72]. A helicoid shape was achieved by heating a ribbon of the material to 130°C to break the hydrogen bonds, clamping it on one end, and twisting the other end and holding the ribbon in this state while cooling to reform the hydrogen bonds. The helicoid would reversibly contract and unwind when the temperature was cycled between room temperature and 100°C (Figure 9c). Similarly, a ribbon with a left‐handed segment and a right‐handed segment joined by a flat segment was prepared. Helicoid and helical fibers of this material can be prepared using meld extrusion [75, 76], and UV‐responsive thermoplastic helicoid and helical fibers were also prepared by incorporating an azobenzene group into the polythiourethane [77].

Another material for chiral shaping consisted of a liquid crystal polymer network with hydrogen‐bonding 6‐(3‐(2‐(acryloyloxy)ethyl)ureido) picolinate (MAEUP) groups and anthracene‐9‐yl acrylate (An‐A) reversible covalent crosslinks [73]. In a procedure similar to the previous example, the hydrogen bonds were broken at 120°C and the ribbon was wound around a cylinder to shape it into a helix, after which it was cooled down to fix the chiral shape (Figure 9d). This ribbon switched from its chiral form to the flat ribbon and back when the temperature was cycled between room temperature and 75°C. As can be seen from the examples above, when mechanical shaping is used the reversible deformation tends to be in the form of unwinding of the helicoid or helix upon lowering the order parameter, but the reverse has also been demonstrated [78]. In this case a weakly crosslinked helix‐shaped fiber with multidomain alignment was prepared using a screw mold, then stretched to align the molecules, and finally the fiber was fully crosslinked (Figure 9e). The resulting straight fiber returned to its helix shape upon heating (Figure 9f).

**FIGURE 10:**
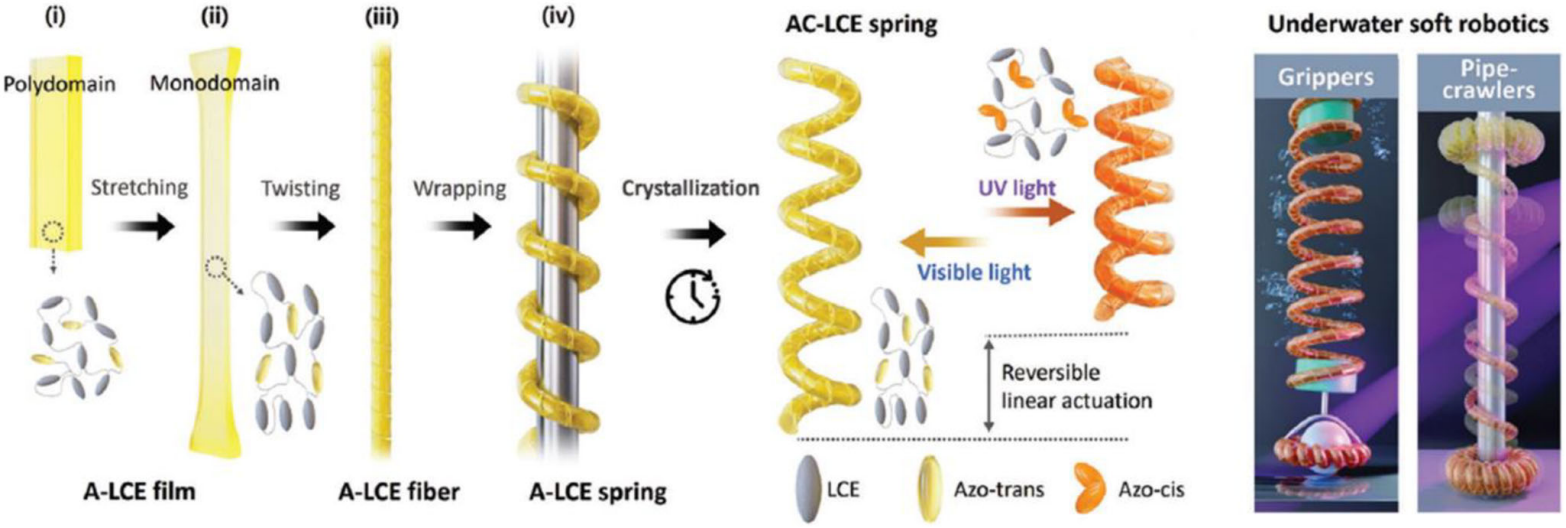
Mechanical shaping technique that involves a two‐step shaping process. In the first step the film is stretched and then twisted. In the second step the twisted fiber is wrapped around a cylinder, and this helix shape is fixed. In this example the fiber contains azobenzene groups that cause the helix to contract upon UV‐irradiation, with various potential applications being demonstrated. Reproduced with permission [81]. Copyright 2024, Wiley‐VCH.

An interesting mechanical shaping technique involves a two‐step process of winding a ribbon or fiber around its central axis, followed by shaping it into a coil (Figure 10) [79, 80, 81]. The ribbon or fiber is stretched to increase its length and align the molecules along the stretching direction. Then the ends are clamped, and the sample is twisted along its long axis. In the next step the twisted sample is wrapped around a cylinder to shape it into a coil, and this shape is fixed. The resulting helices have hierarchical chirality and are either homochiral when the handedness of the twist and the helix are the same or heterochiral when they are opposite, and these behave differently upon actuation. The twist process amplifies the actuation strain of the coils. Chiral shaping and reshaping was also achieved in polysiloxane liquid crystal polymer networks with ester crosslinks and hydroxyl groups [74]. These moieties start to rearrange when the material is heated to 120°C, allowing the material to be shaped. Upon cooling, this shape is fixed. Azobenzene groups were incorporated in the network, and UV‐responsive helicoids and helices were prepared through the shaping process.

### 3.3 Chiral Actuators Made Using 3D Printing

Another technique to prepare liquid crystal networks with chiral shape deformations is 3D printing (often called 4D printing), which allows the preparation of complex 3D objects [82, 83, 84]. The polymer material is extruded from a nozzle that moves along a programmed path, and the liquid crystal molecules align in the printing direction due to shear forces. The object is built in a layer‐by‐layer manner. To achieve chiral deformation in a 3D printed ribbon the bottom half is printed with the alignment director at an acute angle to the long axis of the object, and the other side is printed in the orthogonal direction. This geometry somewhat resembles a ribbon with twisted nematic alignment, with θ determined by the printing direction. These ribbons strongly resemble the previously discussed bilayers that were prepared by combining two liquid crystal polymer films, but here the whole ribbon is created in one step without the need for assembly. This technique also resembles chiral shaping in that the liquid crystals are aligned through shear forces, and the alignment director geometry is better controlled. In a way, 3D printing combines the best characteristics of bilayer assembly and chiral shaping, but it does require specialized equipment and expertise.

**FIGURE 11:**
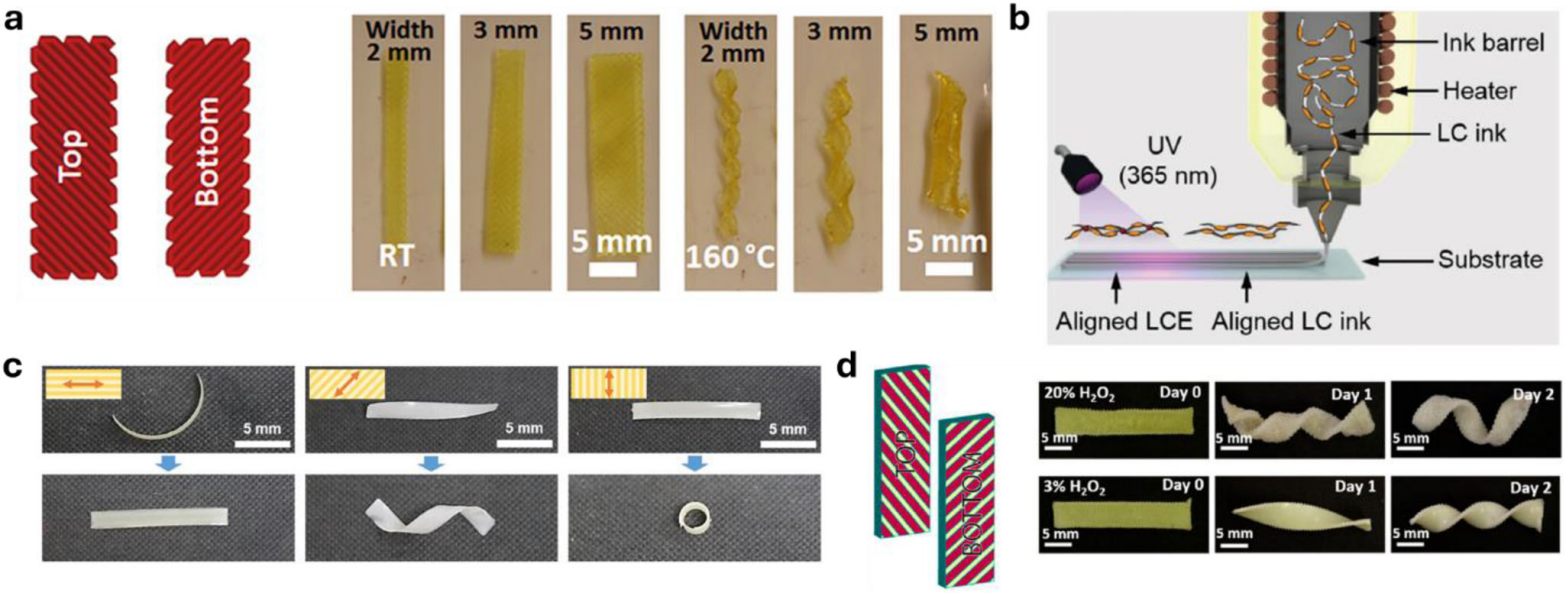
(a) Preparation of chiral heat‐responsive actuators through 3D printing of a main‐chain liquid crystal oligomer. Two layers are printed on top of each other, with the top layer having the alignment director orthogonal to the bottom layer. The chiral shape appears upon heating, with a narrow actuator deforming into a helicoid and a broad one deforming into a helix. Reproduced with permission [85]. Copyright 2017, American Chemical Society. (b) 3D printing setup for preparing moisture‐responsive ribbons from main‐chain liquid crystal oligomers. Printing of the oligomers is followed by a UV crosslinking step to obtain solid actuators. Reproduced with permission [53]. Copyright 2021, Wiley‐VCH. (c) Deformation of various moisture‐responsive ribbons prepared through 3D printing. The ribbons were activated on one side, and only that side swells upon exposure to moisture. Reproduced with permission [53]. Copyright 2021, Wiley‐VCH. (d) Chiral actuators based on oxidation‐responsive main‐chain liquid crystal oligomer. In 20% H_2_O_2_ the ribbon deforms into a helix, but at 3% it deforms into a helicoid. The chiral shapes slowly unwind over time. Reproduced under the terms of the CC‐BY Creative Commons Attribution 4.0 International license [54]. Copyright 2020, MDPI.

In one example of 3D printing, an acrylate‐terminated main‐chain liquid crystal oligomer was printed and cured through UV‐initiated crosslinking (Figure 11a) [85]. By varying the aspect ratio of the objects, they could be made to deform into helicoids or helices, as was previously observed in ribbons with twisted nematic alignment. In a different study, the same material was printed to investigate the effect of θ on the deformation behavior [86]. It was found that larger angles lead to a shorter pitch length in objects that deformed into helicoids. The main‐chain oligomer that could be treated with aqueous HCl to induce water‐responsiveness was also 3D printed (Figure 11b,c) [53]. A single layer with diagonal uniaxial alignment was printed, and to achieve bending the ribbon was treated with the HCl solution on only one side so only that side would expand when the ribbon was immersed in water, causing deformation into a helix. The oxidation‐responsive main chain oligomer was also 3D printed (Figure 11d) [54]. Like in some of the previous examples, the printed rectangular objects consisted of two layers with diagonal, orthogonal alignment directors. Interestingly, the objects deformed into helices when immersed in 20% H_2_O_2_ while deforming into helicoids when immersed in 3% H_2_O_2_. In addition, the helices initially wound tightly and then would slowly unwind over time. Both of these behaviors might be explained by a change in elastic modulus caused by the H_2_O_2_‐induced degradation, but an exact explanation has not been provided.

**FIGURE 12:**
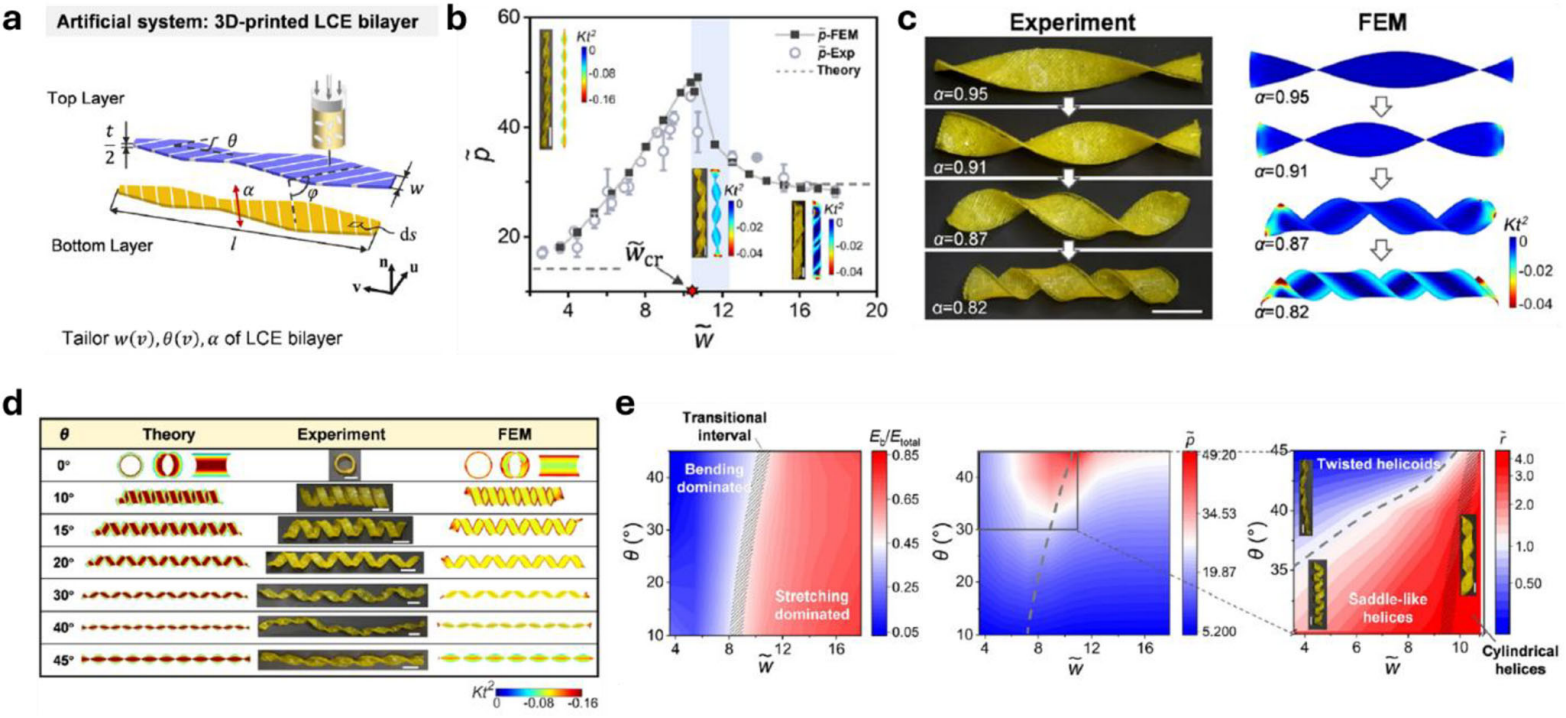
(a) Schematic representation of the 3D printing procedure of bilayer ribbons and the relevant printing parameters. (b) Shape selection of heated ribbons with θ = 45°, ϕ = 90°, and α = 0.8, showing the relation between w̃ p̃. The vertical blue bar indicates the critical values w̃_cr_ where the shape transitions from a helicoid to a helical ribbon. (c) Deformation of a ribbon (θ = 45°, ϕ = 90°, and w̃ = 14.29) as a function of α. The corresponding finite element analysis is presented on the right. (d) Deformation of ribbons (ϕ = 90°, w̃ = 3.75 and α = 0.8) as a function of θ. The corresponding finite element analysis is presented on the right. (e) Phase diagrams showing the combined effect of w̃ and θ on the shape selection of the ribbons at α = 0.8. The red and blue colors show the energies as calculated through finite element analysis. Figures reproduced with permission [87]. Copyright 2025, Elsevier.

An in‐depth systematic study demonstrated that the use of 3D printing enables a great deal of control over the deformation behavior of the printed chiral ribbons (Figure 12a) [87]. The effect of several printing parameters on the deformation was studied. Some key parameters that were defined in the study were the offset angle θ, the twist angle ϕ, the dimensionless width w̃ (defined as w/t, where w the ribbon's width and t is its thickness), and the shrink ratio α (defined as the heat driven contraction of the material I/I_0_, where I is the length at the current temperature and I_0_ is the initial length). The experiments were supported by finite element analysis. As in previous studies, the aspect ratio was found to determine the shape selection, with narrow ribbons forming helicoids and broad ribbons forming helices (Figure 12b). The dimensionless pitch p̃ *(= p/t)* was found to increase with increased w̃ for helicoids, but after the helicoid to helical ribbon transition p̃ decreased with increased w̃. The effect of α on the shape selection was also studied (Figure 12c). At the fixed parameters θ = 45°, ϕ = 90°, and w̃ = 14.29 the ribbons were found to adopt a helicoid shape at high values of α, but transition into a helical ribbon at lower values of α. The effect of θ was studied using ribbons with parameters ϕ = 90°, w̃ = 3.75 and α = 0.8. As expected, at low values of θ helical ribbons were observed while at values of θ close to 45° helicoids were observed (Figure 12d). The combined data allowed for the creation of phase diagrams to show the effect of both w̃ and θ on the shape selection (Figure 12e).

## 4 Applications of Chiral Liquid Crystal Actuators

### 4.1 Functional Actuators Inspired by Plant Tendrils and Flowers

As mentioned in the introduction, plants often employ chiral shapes in their tendrils and leaves. The helical deformations of liquid crystal polymer ribbons are similar to these chiral shapes in plants, and a parallel is sometimes drawn between the two [11, 88]. Plant tendrils curl up into helices, and they often reverse handedness halfway along their length with a small straight section between the two chiral sections. These features have been incorporated in plant tendril mimics based on liquid crystal polymers, as will be discussed here.

**FIGURE 13:**
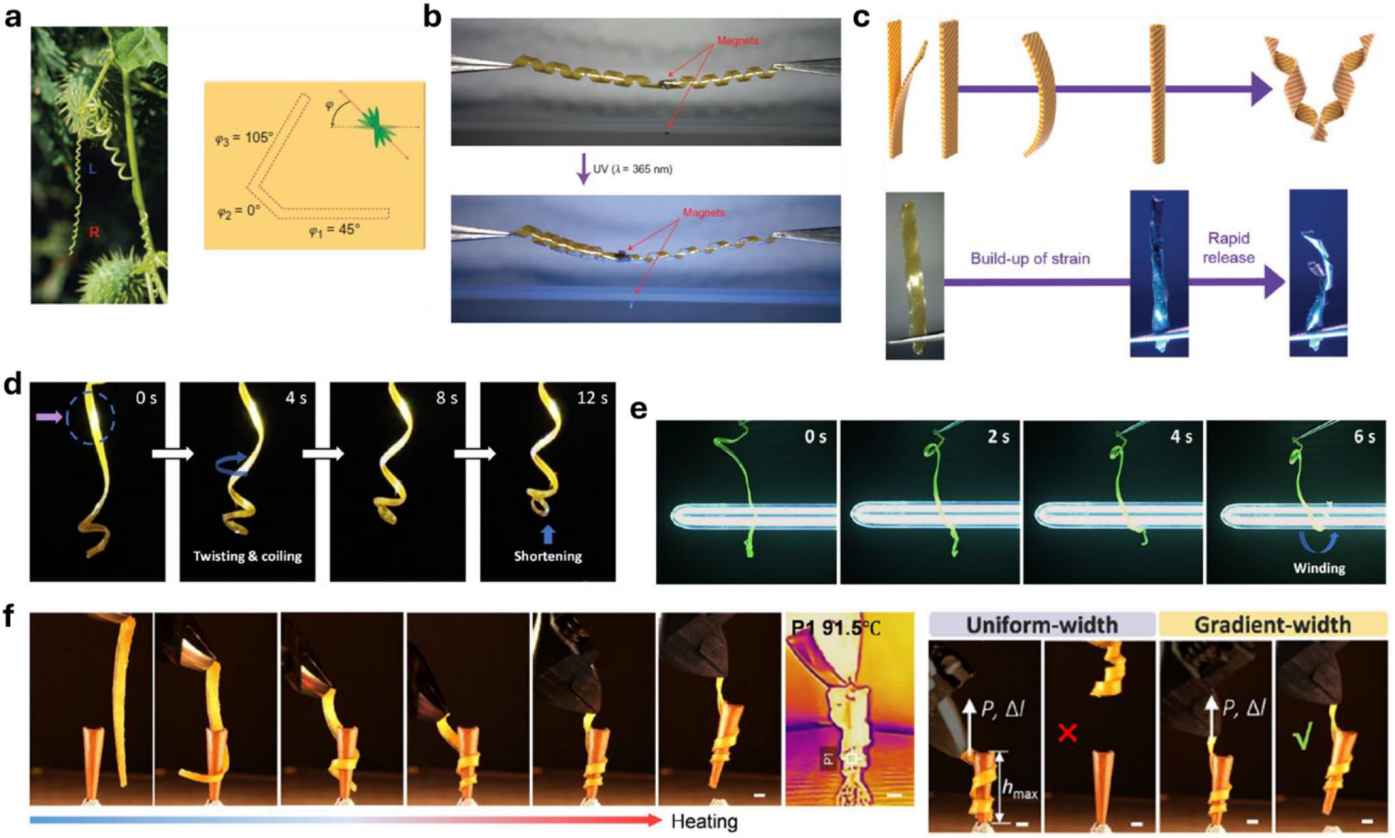
(a) Design of a plant tendril mimic based on a liquid crystal polymer network with twisted nematic alignment. A single ribbon was cut with segments at three different angles with respect to the alignment director. These segments deformed into a shape consisting of a left‐handed helix and a right‐handed helix joined by a kink. Reproduced with permission [58]. Copyright 2014, Springer Nature. (b) Deformation of the plant tendril mimic upon UV irradiation. The right‐handed helix unwinds while the left‐handed helix winds, which moves the kink towards the left‐handed helix. A magnet is placed at the kink which moves with it and can in turn move a second magnet. Reproduced with permission [58]. Copyright 2014, Springer Nature. (c) Design and operation of a seedpod mimic. Two enantiomer actuators with a bar pattern were combined to form the seedpod. Upon UV irradiation the device initially bends slightly, after which is suddenly snaps open. Reproduced under the terms of the CC‐BY Creative Commons Attribution 4.0 International license [89]. Copyright 2017, Wiley‐VCH. (d) UV‐responsive helix prepared through extrusion rolling. The helix contracts upon prolonged UV exposure. Reproduced under the terms of the CC‐BY Creative Commons Attribution 4.0 International license [90]. Copyright 2025, MDPI. (e) A biomimetic tendril made by extrusion molding that rapidly winds around a UV light tube. Reproduced under the terms of the CC‐BY Creative Commons Attribution 4.0 International license [90]. Copyright 2025, MDPI. (f) A 3D printed bilayer ribbon with a tapered width wrapping around a conical object. Due to the tapering the pitch of the helix exactly follows the conical shape, while a uniform width ribbon fails to tightly wrap around the same object. Reproduced with permission [87]. Copyright 2025, Elsevier.

In one case, responsive plant tendril mimics were prepared using the light‐responsive winding, unwinding and helix inversion material that was discussed in part 2 of this review (Figure 5) [58, 59]. As explained there, ribbons cut from this polyacrylate material immediately deformed into helices after being cut from the film, and would then either wind further or unwind upon UV exposure, with the cutting angle with respect to the alignment determining which behavior was observed. This property of the material was used to create a ribbon with a left‐handed helix on one side and a right‐handed helix on the other side, which were linked by a kink, by cutting different sections of the ribbon at different angles (Figure 13a). Upon exposure to UV light the right‐handed helix would unwind, and the left‐handed helix would wind up (Figure 13b). When the whole ribbon was irradiated at once, the kink would be moved towards the left‐handed side. By switching the UV irradiation on and off, a repeating lateral motion could be achieved, and by attaching a magnet to the kink and have this magnet interact with a second magnet, an up‐and‐down shuttling motion could be created in this second magnet. This demonstrates that work can be extracted from these ribbons purely by cyclical UV irradiation.

A similar materials system was used to create UV responsive seedpod mimics (Figure 13c) [89]. In this case, polymer films with an alternating bar pattern were prepared using photomasks, by photopolymerizing some bars with UV light and the others with visible light. The bars polymerized with UV light had a lower nematic order due to the trans‐to‐cis isomerization of the azobenzene. Films cut at +45° and ‐45° deformed into helices of opposite handedness upon removal from the alignment cells, and two of these enantiomer helices were combined to form the seedpod. Upon UV irradiation, the initially flat artificial seedpod shows some bending until the two films suddenly detach from each other and twist into helices, causing the pod to pop open rapidly.

A different plant tendril mimic was prepared by mechanical shaping of a main‐chain liquid crystal polymer network that deformed from straight into a helix upon IR irradiation (as previously shown in Figure 9e,f) [78]. A weakly crosslinked coil was prepared using a screw mold, and this coil was stretched into a straight fiber and further crosslinked to fix the stretched state. The material was doped with graphene to improve its photothermal responsiveness. Upon IR irradiation the straight fibers deformed into coiled tendrils. These tendrils also showed various kinds of self‐oscillation upon irradiation. A UV light‐responsive tendril was prepared using extrusion rolling of main‐chain liquid crystal polymers with azobenzene groups to mimic tropism in plants [90]. The un‐crosslinked polymer was extruded from a syringe and collected on the outside of a rotating second syringe barrel to create a coil shape. This coil was collected, stretched, and cured through UV crosslinking. As expected, the straight tendril would deform into a coil upon exposure to UV irradiation (Figure 13d,e).

Printing was also used to create plant tendril mimicks. In one such study, to more closely mimic the structure of plants, tendrils with a core‐sheath structure were prepared [91]. The printed main‐chain oligomers contained 2,6‐bis(benzimidazolyl)‐4‐pyridine moieties that functioned as noncovalent crosslinkers by forming coordination complexes with zinc ions for a higher viscosity, which enhanced the printability and alignment of the material. Increased shearing of the surface of the fibers during 3D printing, as well as anisotropic deswelling at the surface during solvent evaporation, caused the fibers to have a structure with a well aligned sheath enclosing a less aligned core. Due to a difference in anisotropic deswelling between the top and bottom of the film, there was a difference in order between these two sides, causing the fibers to have dorsal‐ventral asymmetry. Upon heating these fibers reversibly formed helices, an effect which was assumed to be caused by a larger contraction of the sheath compared to the core as well as well as a larger contraction of the dorsal side compared to the ventral side. However, it is not clear what determined the handedness of the helices. Reshaping the fibers at high temperature was possible due to the noncovalent crosslinkers, which allowed the programming of complex shape deformations. A different study explored the use of 3D printed bilayer ribbons with a tapered widths as plant tendril mimicks [87]. Phase diagrams that relate the aspect ratios and offset angles of the ribbons to the shape selection (see Figure 12e) were used to design the desired helical shapes. As the pitch of the spiral ribbons changes as a function of aspect ratio, a tapered ribbon will have a gradual variation in pitch upon heating. This was used to design a ribbon that selectively wraps around a conical object (Figure 13f).

**FIGURE 14:**
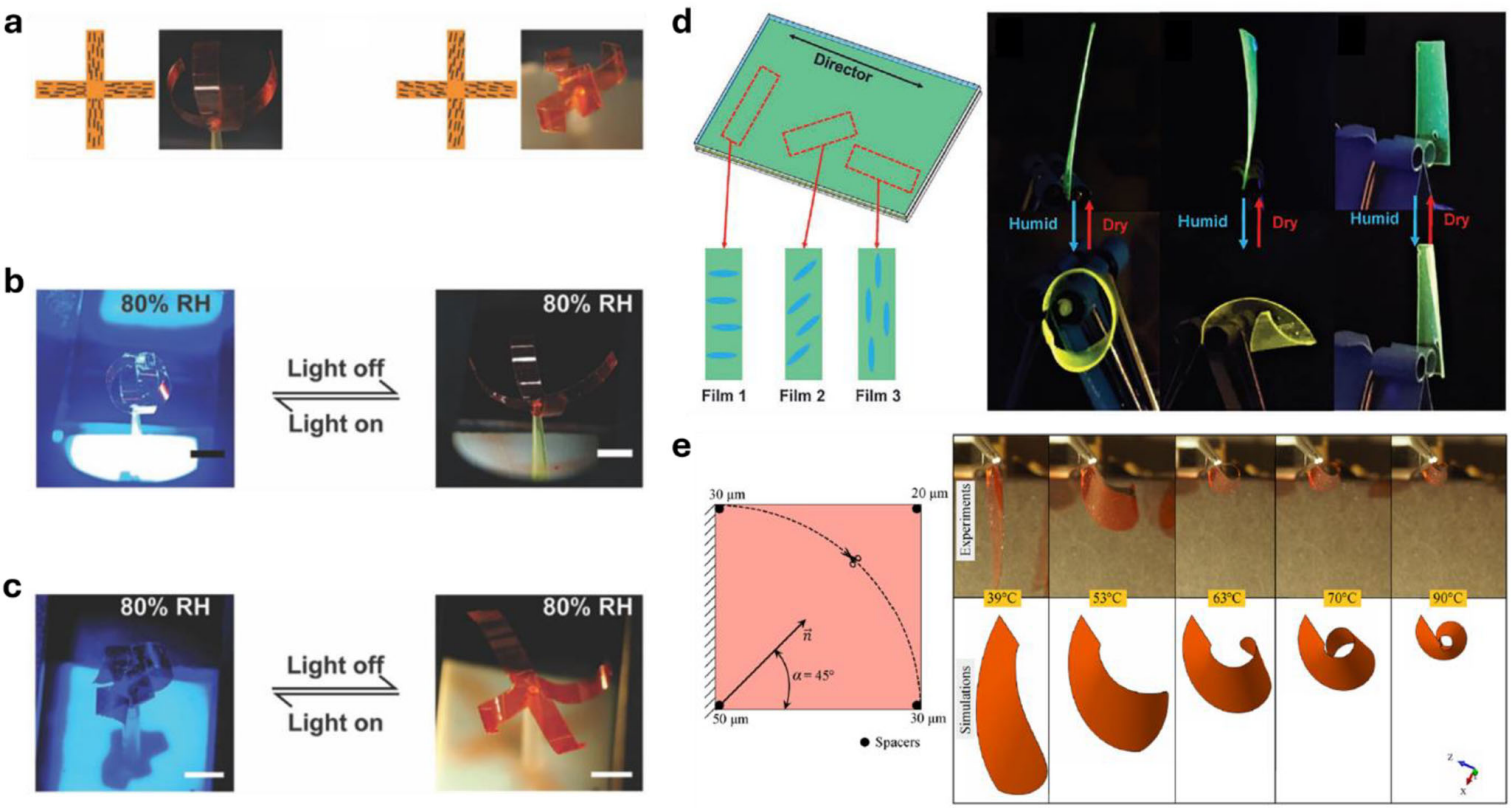
(a) Biomimetic flower based on liquid crystal polymer ribbons. The four petals of the flower can have a parallel or offset alignment director, which leads to a straight or twisted shape, respectively. Reproduced with permission [92]. Copyright 2018, Wiley‐VCH. b) Actuation behavior of the straight biomimetic flower upon UV exposure in high humidity. Reproduced with permission [92]. Copyright 2018, Wiley‐VCH. c) Actuation behavior of the twisted biomimetic flower upon UV exposure in high humidity. Reproduced with permission [92]. Copyright 2018, Wiley‐VCH. d) Biomimetic flower petals made from a bilayer of a liquid crystal network and a water‐responsive polyacrylate film. Swelling of the polyacrylate causes the petal to bend, and the alignment director of the liquid crystal film directs the bending direction. Reproduced with permission [93]. Copyright 2021, Wiley‐VCH. e) Biomimetic Calia Lily flower petal made from a liquid crystal polymer film with splayed alignment and tapered thickness. The alignment on the planar side of the film was 45°. Rolling into a tube upon heating was demonstrated both in experiments and computer simulations. Reproduced with permission [94]. Copyright 2023, Wiley‐VCH.

Besides plant tendrils and seedpods, flower petals have also served as inspiration for the development of biomimetic devices. In one study, the twisted opening and closing of the moon flower was mimicked in a humidity‐gated photo‐actuators based on a liquid crystal polymer network with both humidity‐responsive and photothermal moieties [92]. These flower mimics had four petals, each consisting of a liquid crystal network ribbon. One of the mimics had the alignment director of the liquid crystals parallel to the long axis of the petal ribbons, while the other had the alignment director offset by 10° with respect to the long axis (Figure 14a). The flowers were closed when exposed to UV light or when the humidity level was low and would only be open in the dark at high humidity, in a way similar to some biological plants. The flower with petals with a parallel alignment director closed in a straight way (Figure 14b), while the flower with petals with offset alignment closed in a twisted way (Figure 14c).

Another humidity‐responsive artificial flower was prepared as a bilayer of a liquid crystal network and a water‐responsive polyacrylate film (Figure 14d) [93]. The latter expands when the humidity is high, causing the bilayer to bend, and the aligned liquid crystal network directs this bending in the desired direction. A water‐responsive fluorescent dye was added to the material to incorporate a photonic response in parallel to the shape change. A flower mimic with four petals was prepared, which changed its shape and color when the humidity was raised. Finally, mimics of the Calla Lily flower were developed through finite element analysis and experiments, consisting of sheets with splayed alignment and a tapered thickness that roll up into cones upon actuation (Figure 14e) [94]. The effect of both the alignment director on the planar face of the film and the tapering direction were studied through finite element analysis. It was found that the cone angle increased with the increase in the deviation of the alignment director away from the direction parallel to the edge (or in other words, closer to 45°). A polymer network film with a 45° alignment director and tapering direction was prepared and demonstrated to deform into the desired conical tube upon heating.

### 4.2 Soft Robotics and Other Applications

**FIGURE 15:**
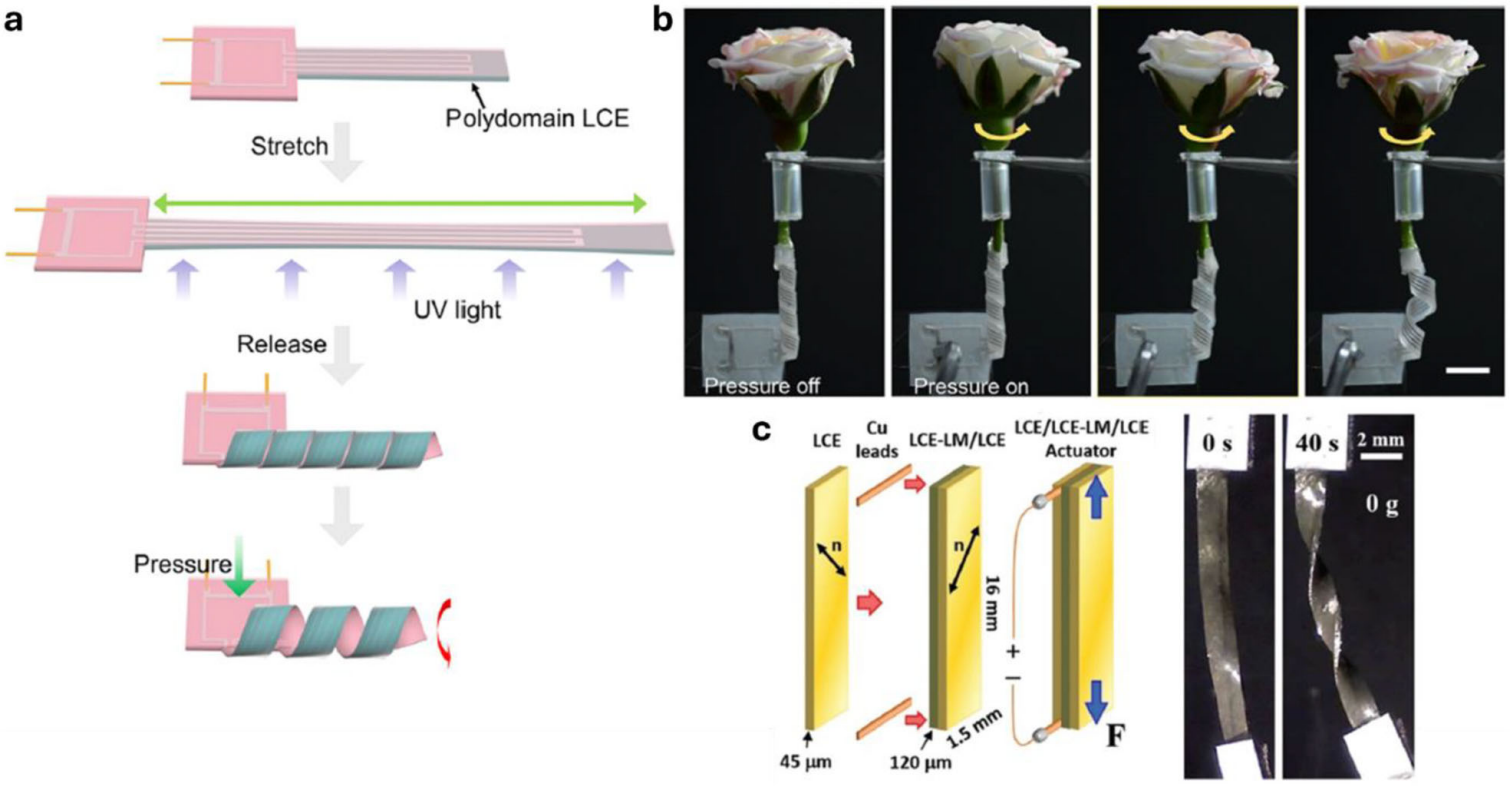
(a) Preparation of a helix actuator with printed liquid metal electrodes for deformation through Joule heating. Liquid metal electrodes were printed onto a polydomain LC film and encapsulated in silicone, and then the LC film was stretched and crosslinked through UV polymerization. When the film was released, it adopted a helix shape. This helix would unwind upon Joule heating, which could be accomplished by putting pressure on the end of the electrodes that wasn't part of the coil. Reproduced with permission [95]. Copyright 2021, American Chemical Society. (b) Rotating flower driven by the unwinding of the responsive helix actuator. Reproduced with permission [95]. Copyright 2021, American Chemical Society. (c) Preparation of a multilayer actuator consisting of two liquid crystal films with a liquid metal film in between, and the deformation from flat to a helicoid upon application of Joule heating. Reproduced with permission [96]. Copyright 2021, Royal Society of Chemistry.

Liquid crystal polymer materials, due to their ability to undergo large reversible shape deformations in response to a stimulus, have been investigated extensively for the development of soft robotic systems. Chiral shape deformations are uniquely suitable for soft robotics applications. The winding and unwinding of helicoid or helix liquid crystal ribbons can be used to induce rotational motion in objects that are attached to one end of the ribbon. In one example, liquid metal electrodes were printed onto a liquid crystal elastomer to enable electrothermal deformation through Joule heating [95]. The liquid crystal elastomer film with the printed liquid metal electrodes, which were encapsulated in silicone, was aligned through stretching followed by crosslinked through UV polymerization (Figure 15a). Upon releasing the film, it deformed into a coil shape, which would unwind upon Joule heating. It is unclear where the geometric misorientation required for the formation of a chiral shape arises from and what determines the handedness of the helix. Interestingly, the Joule heating could be activated by putting pressure on the end of the electrodes that wasn't part of the coil. To demonstrate the use of the actuator in pressure sensitive soft robotics, a flower was attached to the other end of the actuator (Figure 15b). Upon pressing the electrodes, the coil would unwind, causing the flower to rotate. A different device consisted of a multilayer of two liquid elastomer films with a liquid metal film in between them [96]. The alignment of the two liquid crystal films was orthogonal to each other and offset with respect to the long axis of the device. The liquid metal film allowed the application of Joule heating to the device, which caused a deformation into a helicoid shape. This deformation could be used to rotate objects (Figure 15c).

An exciting example of a soft robotics application is in autonomous rolling robots [97]. The first example of a rolling robot based on liquid crystal polymers used an azobenzene‐containing mixture of reactive mesogens to create UV‐responsive polymer films with twisted nematic alignment [98]. These films were cut to obtain ribbons with X‐geometry with θ = 15° at one face. When irradiated with UV light the flat ribbons deformed into helical ribbons, and upon continued irradiation they started rolling. The continuous rolling was caused by the stronger deformation of the parts of the helix that are in the optical path compared to those that are shaded, which led to a net twisting moment and the acceleration of the center of mass. The rolling direction was at an angle with the helical axis, the value of which is directly related to the value of θ.

**FIGURE 16:**
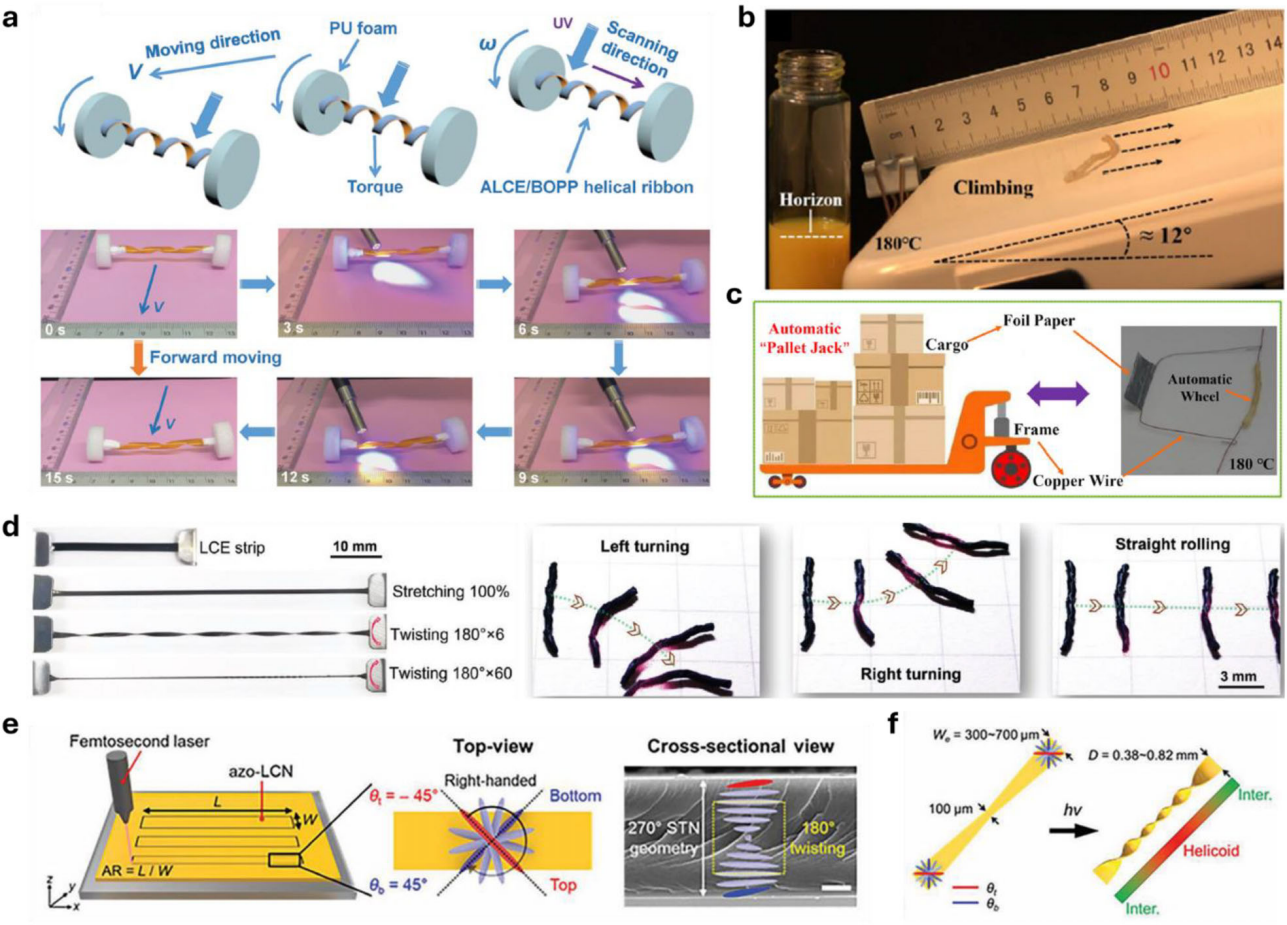
(a) A wheeled soft robot based on a helical liquid crystal actuator with two PU wheels attached to it. The helix deforms upon irradiation with UV light. When a UV light spot is scanning across the helix, the soft robot moves forward. Reproduced with permission [99]. Copyright 2017, Wiley‐VCH. (b) A 3D printed heat‐responsive helical ribbon rolling up a heated incline. Reproduced with permission [100]. Copyright 2021, Elsevier. (c) Cargo transportation by a heat responsive helical ribbon. Reproduced with permission [100]. Copyright 2021, Elsevier. (d) Preparation and operation of IR‐responsive helicoid soft robots. On the left the mechanical shaping of the helicoid is shown. The images on the right show how an IR light spot is used to drive and steer the soft robot. Reproduced with permission [101]. Copyright 2022, Wiley‐VCH. (e) Preparation of liquid crystal polymer ribbons with super‐twisted nematic alignment. The reactive mesogen mixture is filled into alignment cells with orthogonal alignment layers and polymerized, and the ribbons are cut from this film though laser cutting. The super‐twisting is realized by adding additional chiral dopant. Reproduced under the terms of the CC‐BY Creative Commons Attribution 4.0 International license [102]. Copyright 2023, Wiley‐VCH. (f) Tapered ribbons with wide ends and a narrow center. These ribbons have enhanced velocity and steering capabilities. Reproduced under the terms of the CC‐BY Creative Commons Attribution 4.0 International license [102]. Copyright 2023, Wiley‐VCH.

A different device was made using bilayers that were obtained by combining a biaxially oriented polypropylene film and a liquid crystal polyester with azobenzene groups that was prepared through hot pressing and uniaxially aligned by stretching [99]. The bilayers were shaped into a helix by wrapping them around a cylinder at elevated temperature and allowing them to cool in this state. The liquid crystal film could either be on the inside or on the outside of the helix and this changed the device's response to UV irradiation: when the LC film was on the inside the helix would wind tighter, and when it was on the outside the helix would unwind. By irradiating at an oblique angle and scanning the illumination spot from left to right, the helix could be caused to continuously roll, with helices with the LC film on the inside rolling away from the light source and helices with the LC film on the outside rolling towards it. The handedness of the bilayer helix did not seem to have an effect on the light‐driven rolling behavior. By adding a wheel to each end of the ribbon, a light‐driven vehicle was constructed (Figure 16a). A different rolling soft robot was prepared through 3D printing of a main‐chain liquid crystal polymer [100]. The two sides of the actuator had orthogonal alignment directors, so the initially flat ribbon deformed into a helix or helix upon heating. When the helix ribbon was placed on a heated plate it started rolling. The robot was shown to be able to surmount obstacles, climb hills (Figure 16b) and even transport cargo (Figure 16c).

Another rolling robot was prepared from a main‐chain liquid crystal polyester, which contained furan side groups that could be crosslinked through a Diels‐Alder reaction using a bismaleimide crosslinker [79, 80]. Carbon nanotubes were mixed in to the material as a photothermal agent. Twisted fibers of this material were made by heating a cylindrical sample of the material to break the crosslinks, followed by stretching to shape it into a fiber, and then fixing one end and twisting the other end. When heated through IR irradiation, these fibers spontaneously started rolling. The fibers could roll up inclines and through sand and across the surface of water. The twisted fibers could be further shaped into helices with hierarchical chirality by wrapping them around a cylinder. Homochiral helices reversibly contracted on heating and extended on cooling, while heterochiral helices reversibly extended when heated and contracted when cooled, which is due to the untwisting of the twisted fiber upon heating. Both types of helices would roll when exposed to IR irradiation, though the deformation behaviors were different. The rolling upon irradiation resulted from torque that is created as the two ends of the roller are bending upward while an in‐plane curvature forms, which displaced the center of gravity.

IR‐responsive rolling soft robots were also prepared by incorporating an IR absorbing monomer derived from croconaine into a main‐chain liquid crystal polymer network [101]. Ribbons of this LC material were mechanically shaped by twisting into helicoids (Figure 16d). A rectangular IR spot was used to irradiate specific areas of the soft robot to move and steer it along various trajectories. A different study was conducted to improve the steerability and agility of the rolling soft robots [102]. The films were made in alignment cells using a reactive mesogen mixture containing azobenzenes, and the ribbons were cut using laser cutting. Compared to previous rolling robots, a notable difference was the use of super‐twisted nematic alignment with a 270° rotation of the alignment director along the film's thickness (Figure 16e). In this geometry the alignment directors at the two faces of the film were still orthogonal to each other, but an additional 180° is present in the middle layer that a film with 90° twisted alignment would not have. This additional 180° twist neutralizes rotational stress and leads to a straighter helical axis and faster elastic recovery. By varying the aspect ratio, both helical ribbons and helicoids were produced, and in UV‐actuated rolling experiments the latter were found to roll straight while the former deviate off course. The velocity and steering capabilities of the helicoids were further improved by cutting them into a center‐tapered shape with wide ends and a narrow center (Figure 16f).

Besides rotation and rolling, chiral deformation has also been utilized in the development of other soft robotics systems. One example of using chirality in soft robotics is an oscillating film with flexural‐torsional movement to mimic the movement of insect wings [103]. Oscillation in liquid crystal polymer films can be achieved by irradiating a photo‐responsive uniaxially aligned liquid crystal polymer ribbon from one side. In initial work, the alignment director of the liquid crystals was parallel to the long axis of the ribbon, which lead to bending deformation due to contraction along the long axis on the irradiated side. By aligning the director off‐axis, the intended flexural‐torsional oscillation was realized. The deformations of twisted nematic ribbons can be further expanded by confinement [104]. For example, the ends of ribbons with different alignment offset angles, lengths, and widths can be joined to form loops that deform upon heating in various ways, including plectonemic supercoiling, toroidal supercoiling, overcurved buckling, bent tape spring, twisted tape spring, bent and twisted tape spring. In addition, attaching both ends of these ribbons to solid fixtures causes them to fold into complex shapes upon heating. These hierarchical deformations can be used in various soft robotic systems. Another interesting concept is the assembly of multi‐component soft robots containing various chiral actuators [105]. Main‐chain liquid crystal polymer films with terminal acrylate groups were welded by pressing them together and performing thermal polymerization. The chiral shapes were created through mechanical shaping, and various multi‐component soft robots containing chiral ribbons were assembled. For example, a rotating fan like structure consisting of an unwinding helical ribbon to induce rotation with three blades at the end was constructed.

Semi‐crystallinity, which leads to significant mechanical enhancement, was achieved in main‐chain liquid crystal polymers by using monomers and chain extenders with long aliphatic chains [81]. The helix actuators were prepared through the previously mentioned two‐step technique for achieving hierarchical chirality, where a strip of the material was stretched to align the molecules, then twisted, and finally wrapped around a cylinder. The helix was stored in the wrapped state at room temperature to allow crystallization to fix the shape. As seen before, homochiral helices reversibly contract upon UV‐irradiation while heterochiral helices reversibly extend. This effect was mainly photochemical and only partially photothermal in nature, which allowed the actuators to work underwater. Soft grippers and pipe crawlers were demonstrated which consisted of an expanding heterochiral body and one or two contracting homochiral gripping head(s) (Figure 10).

An interesting application with potential as a medical device was demonstrated using a programmable liquid crystal polymer ribbon with a helix shape as an insertable blood vessel patch [106]. A ribbon of a densely crosslinked liquid crystal network with azobenzene moieties, with the unidirectional alignment director at a 30° angle with the long axis of the ribbon, was irradiated with UV light to produce a metastable state with residual photo‐stress. When heated to body temperature (37°C) this residual stress was released and the film deformed into a densely wound helix that was narrow enough to be inserted into a blood vessel. The helix would then slowly unwind over a 10 min period to a radius similar to that of the blood vessel to fit snugly into the confined space. Another intriguing and creative application for ribbons with flat to helix transitions is reversible aggregation [56]. Ribbons with twisted nematic alignment were suspended in silicone oil and the suspension was heated to trigger the deformation. The shape change of the ribbons causes them to mechanically entangle upon a temperature increase. The entangled aggregate behaves as a solid‐like material with its own mechanical properties. The effect of the ribbon length and the offset angle were investigated. It was found that offset angles of 0° or 10° lead to aggregation, while an offset angle of 45° does not. This is because ribbons with a 0° offset angle bend and ribbons with a 10° offset angle form helices, which both facilitate entanglement, while the ribbons with a 45° offset angle form helicoids which do not entangle.

## 5 Conclusions and Outlook

To summarize, shape morphing from a flat ribbon into a 3D chiral shape requires a combination of mechanical anisotropy, geometric misorientation, and asymmetry between the two faces of the ribbon. The first requirement is straightforward to meet in liquid crystal polymer networks with monodomain alignment, as these materials are inherently mechanically anisotropic. This makes liquid crystals the top choice for achieving chiral morphing. The second requirement can be met by making sure that the alignment director at one or both surfaces is at an acute angle with the long axis of the ribbon. In monolithic ribbons, the third requirement can be met using twisted or splayed alignment, or by chemically induced asymmetry between the two faces, while in bilayers the asymmetry is provided through a mismatch in mechanical properties between the two layers. Such bilayers can be assembled by combining a liquid crystal film with a non‐liquid crystal film and making use of the stimuli‐responsiveness of either or both layers to induce shape morphing, or by combining two liquid crystal film with mismatched alignment directors to create an effect similar to that in monolithic ribbons with twisted nematic alignment. In all these cases the initial ribbon is flat and nonchiral, but the chirality is initially “hidden” inside the geometric misorientation, even if the material lacks chirality on the molecular and/or supramolecular level, to subsequentially be “revealed” when the stimulus is applied. By including unreactive mesogens in the polymerization mixture, irreversible chiral morphing can be induced through deswelling. Unwinding and handedness reversal can be then achieved in these chiral ribbons by lowering the liquid crystal order using heating or UV irradiation.

Alternatively, mechanical shaping can be used to create materials who's ground state is a chiral shape and which undergo shape morphing upon being exposed to a stimulus, the exact nature of which is determined by the alignment of the mesogens. This methodology has increased potential for upscaling and enables the possibility of preparing chiral fibers instead of ribbons, which would be difficult to do using classical alignment techniques. However, the alignment geometry is often not very well known. Mechanical shaping requires materials that are viscous enough to stay in a certain shape, at least temporarily, and can then be fixed in this shape by, for example, crosslinking or crystallization. An interesting methodology is to first create a weakly crosslinked helical fiber using a chiral screw mold, followed by mechanical stretching into a straight fiber and further crosslinking to fix the nonchiral shape. Analogous to the previously discussed flat ribbons, these straightened fibers possess a hidden chirality in their molecular alignment that manifests in a 3D shape deformation upon heating, as the fiber morphs from straight into helical. 3D printing combines the shear flow alignment of mechanical shaping with precise control over the alignment director geometry. This technique is usually used to create liquid crystal polymer bilayers that perform flat‐to‐chiral shape morphing upon application of a stimulus.

Chiral shapes are ubiquitous in nature, and the many reports on chiral plant mimics based on liquid crystal polymers demonstrate that scientists often find inspiration in the biological world. Reports of artificial plant tendrils, seedpods and flower leaves show the critical importance of the chirality that liquid crystal polymers enable to further the progression of functional biomimetics. Besides biomimetics, the field of soft robotics is the main driver for the development of chiral liquid crystal actuators. Stimuli‐responsive helicoids and helices can be used to turn temperature variations into rotational motion, while rolling soft robots turn temperature‐ and light‐based stimuli into unidirectional movement. Helical ribbons and fibers can be deployed in tube‐shaped environments such as pipes, but also inside blood vessels, the latter opening up promising biomedical applications. Tapering of the width of the ribbons has been used in several of the demonstrated devices as a method to further refine the shape morphing, and using this method has even allowed morphing into 3D spiral shapes, which was previously difficult to accomplish.

An intriguing observation is that in most of the described applications the actual handedness of the ribbons or fibers is of little to no importance, and most of the devices work equally well with left‐handed and right‐handed shapes. It seems that, when compared to nonchiral stimuli‐responsive actuators that simply contract, extend or roll up, the unique properties of helicoid and helix shapes themselves are often considered of greater importance than the fact that these shapes possess chirality. It does make one wonder if handedness should be considered more often when new applications are designed, which could perhaps open up routes towards new applications that were not previously envisioned. In rotating soft robotic components, the handedness of the rotating element determines if the rotation is clockwise or counterclockwise, which can matter for certain applications. A more advanced possibility could be in soft robotic communication. For example, devices that have chiral elements that recognize chiral shapes of the same handedness and reject the opposite handedness, or vice versa, could be interesting for applications in self‐learning robotics, communication, and security.

Another interesting observation is that while the twisted nematic alignment is ubiquitously used to achieve chiral shape morphing, the splayed alignment is almost never encountered in the literature regarding chiral actuators. While it can be appreciated that films with splayed alignment are significantly more difficult to make, it does raise the question if this leaves a potentially interesting and useful avenue for discovery unexplored. Hopefully, the newer techniques to prepare liquid crystal actuators, such as 3D printing, can be deployed to open up this mostly untouched research direction.

The collection of research work presented here should make it clear that the research interest in chiral shape morphing of liquid crystal networks is rapidly increasing. While initially the literature on this subject consisted mainly of a handful of important theoretical and fundamental studies, in recent years the scope of the topic has significantly broadened to include biomimetics, biomedical applications, and soft robotics. Further development of novel chiral devices, preparation techniques and potential applications will continue when going forward into the future, and hopefully this review can be a guideline towards that future. After all, considering how ubiquitous chirality is in the world around us, and how important it is to our life and wellbeing, it is only sensible to be inspired by and to incorporate this fascinating aspect of reality into the promising field of liquid crystal polymer research as much as possible.

## Author Contributions

**Laurens T. de Haan**: Writing – review and editing, Writing – original draft.

## Conflicts of Interest

The authors declare no conflicts of interest.

## Data Availability

Data sharing not applicable to this article as no datasets were generated or analysed during the current study.
